# Early and Late Steps of Quinine Biosynthesis

**DOI:** 10.1021/acs.orglett.1c00206

**Published:** 2021-02-24

**Authors:** Francesco Trenti, Kotaro Yamamoto, Benke Hong, Christian Paetz, Yoko Nakamura, Sarah E. O’Connor

**Affiliations:** Department of Natural Product Biosynthesis, Max Planck Institute for Chemical Ecology, Hans-Knöll-Straße 8, 07745 Jena, Germany

## Abstract

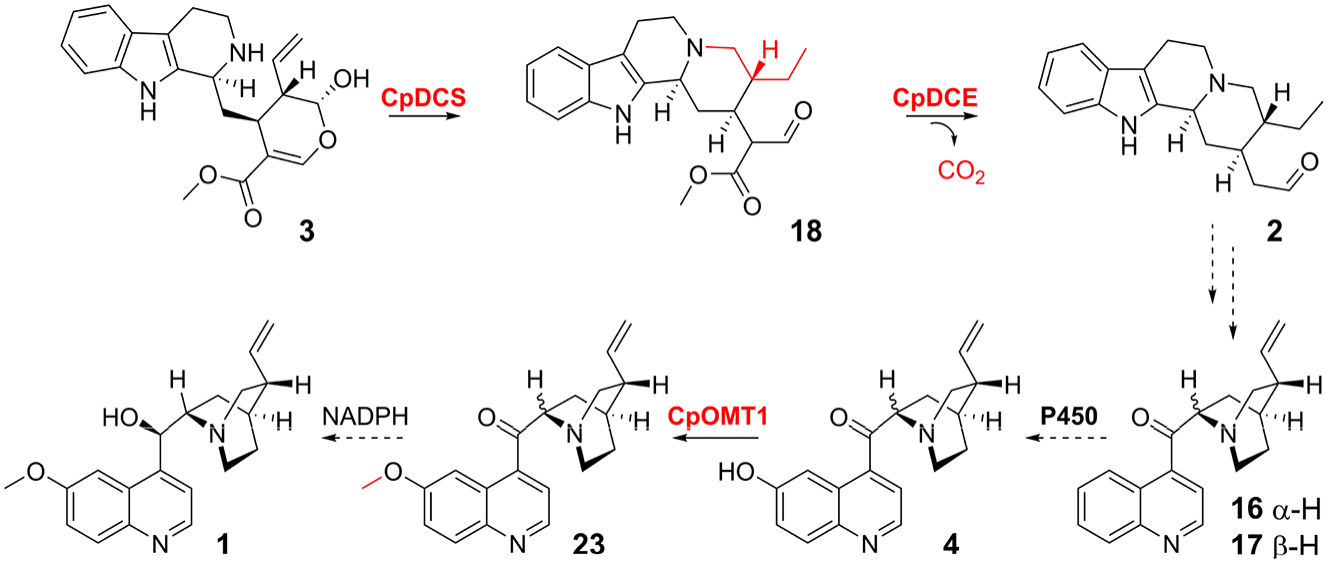

The enzymatic basis for quinine **1** biosynthesis was investigated. Transcriptomic data from the producing plant led to the discovery of three enzymes involved in the early and late steps of the pathway. A medium-chain alcohol dehydrogenase (CpDCS) and an esterase (CpDCE) yielded the biosynthetic intermediate dihydrocorynantheal **2** from strictosidine aglycone **3**. Additionally, the discovery of an *O*-methyltransferase specific for 6′-hydroxycinchoninone **4** suggested the final step order to be cinchoninone **16/17** hydroxylation, methylation, and keto-reduction.

Quinine **1** is an alkaloid produced by *Cinchona* trees of the Rubiaceae family, historically used as an antimalarial drug and as a flavor ingredient in beverages such as tonic water.^1−3^ Although the total synthesis of quinine has been achieved,^4^ the enzymes of this biosynthetic pathway remain undiscovered. In the present work, we combined metabolomics and transcriptomics data from different tissues of *Cinchona pubescens* to identify genes involved in the biosynthesis of quinine and related compounds. We report the discovery of the enzymes required to form the early biosynthetic intermediate dihydrocorynantheal **2** from strictosidine aglycone **3** via reduction and esterase-triggered decarboxylation. Additionally, we identified an *O*-methyltransferase specific for 6′-hydroxycinchoninone **4**, suggesting a preferred order for the late steps of quinine biosynthesis *in planta*.

Methanolic extracts from roots, stems, and leaves in different developmental stages of the quinine-producing plant *C. pubescens* were subjected to untargeted metabolomic analysis. Roots and stem presented a similar metabolomic profile, showing the accumulation of quinine **1** as well as the derivatives quinidine **5**, cinchonine **6**, and cinchonidine **7**. Notably, dihydroquinine **8**, dihydroquinidine **9**, dihydrocinchonine **10**, and dihydrocinchonidine **11**, derivatives in which the terminal methylene group is reduced, were also observed in roots and stem. Leaves contained **6**, **7**, **10**, and **11** but not the methoxy derivatives **1**, **5**, **8**, and **9** (Figure 1, Figure S1). These compounds were identified by comparison with purchased standards. RNA was prepared from the same organs and was subjected to sequencing.

**Figure 1:**
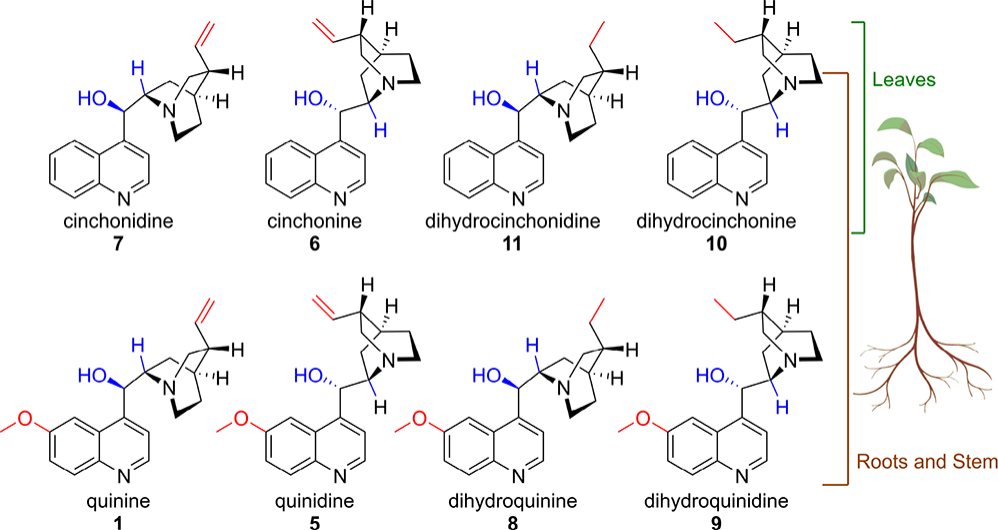
Metabolomic analysis of *Cinchona* alkaloids in plant roots, stems, and leaves.

To identify biosynthetic gene candidates from the RNA-seq data set, we first developed a hypothesis for the enzymatic transformations required for quinine biosynthesis based on previously reported feeding studies in *Cinchona* spp. as a starting point. Hay et al. conclusively demonstrated through feeding of radiolabeled secologanin and tryptamine that **1** is a derivative of strictosidine **12**, which is the central intermediate in the biosynthesis of all monoterpene indole alkaloids (MIAs) including aspidosperma, iboga, and quinoline scaffolds.^5,6^ Battersby et al. used evidence from *in planta* tracer experiments to propose the early biosynthetic intermediate corynantheal **13**.^7^ Because nearly all MIA pathways begin with the deglycosylation of strictosidine **12**, we proposed that strictosidine aglycone **3** is reduced to form corynantheine aldehyde **14**, which can be de-esterified to enable decarboxylation to form **13** (Scheme 1). Battersby proposed that corynantheal **13** could be converted through a series of redox reactions to cinchonaminal **15**.^7a^ The indole moiety of cinchonaminal **15** could then rearrange to the quinoline structure found in cinchoninone **16** and cinchonidone **17**, again presumably through redox transformations (Scheme 1).^7b^ Note that for simplicity, we refer to this mixture of **16** and **17**, which are epimers, as cinchoninone **16/17**. Isaac et al. observed the NADPH-dependent keto-reduction of the desmethoxy derivatives **16/17** to the epimers cinchonine **6** and cinchonidine **7** using protein extracts from *C. ledgeriana* suspension cultures (Scheme 1).^8^ Finally, the hydroxylation and methylation of cinchonine **6** and cinchonidine **7** would subsequently yield quinidine **5** and quinine **1**, respectively. Note that the order of reduction, hydroxylation, and *O*-methylation to go from **16/17** to **1** is not known.

**Scheme 1:**
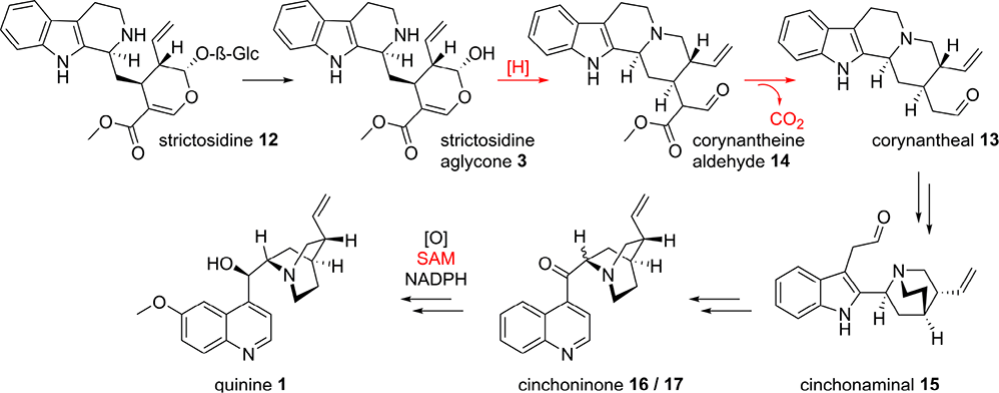
Key Intermediates on the Hypothetical Pathway of Quinine Biosynthesis Steps elucidated in this study are highlighted in red.

On the basis of this biosynthetic hypothesis, RNA-seq data were mined for oxidases, reductases, esterases, and *O*-methyltransferases. We hypothesized that quinine biosynthetic genes would be evolutionarily related to other MIA biosynthetic enzymes. Therefore, we limited our search among these classes of enzymes to orthologues of MIA biosynthetic genes from other plant species that have been previously reported (Figure S2).^9,10^ Specifically, we searched the *C. pubescens* RNA-seq data for orthologues of geissoschizine synthase, a medium-chain alcohol dehydrogenase (MDH) that reduces strictosidine aglycone in vinblastine biosynthesis in *Catharanthus roseus*;^11^ orthologues of tabersonine-16-hydroxylase, a cytochrome P450 (P450) that hydroxylates an MIA in *C. roseus*;^12a^ orthologues of 16-hydroxytabersonine *O*-methyltransferase (OMT), a methyl transferase that methylates an aromatic hydroxyl group of an MIA;^12b^ and orthologues of polyneuridine aldehyde esterase from *Rauwolfia serpentina*, which catalyzes hydrolysis of the methyl ester of an MIA, triggering a spontaneous decarboxylation (Figure S2).^13^ We reasoned that reduction catalyzed by a geissoschizine synthase orthologue followed by hydrolysis and decarboxylation catalyzed by a polyneuridine aldehyde esterase orthologue would yield corynantheal **13** from strictosidine aglycone. We further hypothesized that orthologues of tabersonine 16-hydroxylase and 16-hydroxytabersonine *O*-methyltransferase could be responsible for the late-stage methoxylation of the quinoline scaffold, namely, the conversion of cinchonidine **7** to quinine **1**. We focused on transcripts that displayed high expression levels in stem and roots, correlating with the presence of **1** in those organs. Ultimately, 16 MDHs, 6 esterases, 20 P450s, and 7 OMTs were cloned and successfully expressed in *E. coli* (MDHs, OMTs, esterases) or *S. cerevisiae* (P450s).

All 16 MDH/geissoschizine synthase orthologue candidates cloned from *C. pubescens* were tested in combination with *C. roseus* strictosidine glucosidase (CrSGD) using strictosidine **12** as a substrate to generate strictosidine aglycone **3**.^14,15^ A single MDH completely consumed the strictosidine aglycone **3** starting material, with the major product **18** having a detected mass of *m*/*z* 355.20 (Figure 2, Figure S3). Although the expected *m*/*z* of corynantheine aldehyde **14** is *m*/*z* 353.19, we speculated that the product of this enzyme could be the over-reduced product dihydrocorynantheine aldehyde **18**, the putative biosynthetic intermediate for the dihydro-quinine compounds **8**–**11**. Because metabolomic analysis showed that members of the dihydro-series of quinine-related compounds were highly abundant in these plant tissues, it was not surprising to find a reductase capable of catalyzing this reaction (Figure 1). Unfortunately, efforts to purify this compound resulted only in decomposition. However, when this enzymatic product was further incubated with one of the six polyneuridine aldehyde esterase orthologues, a new broad peak with *m*/*z* 297.20 was observed. This molecular weight is consistent with the structure of dihydrocorynantheal **2**, the product of methyl ester hydrolysis and the subsequent decarboxylation of **18** (Figure 2). Approximately 1 mg of this enzymatic product was purified and subjected to full NMR characterization, which validated the predicted structure (Figures S4–S26, Table S3). Therefore, we named the MDH DihydroCorynantheine aldehyde Synthase (CpDCS) and the esterase DihydroCorynantheine aldehyde Esterase (CpDCE).

**Figure 2:**
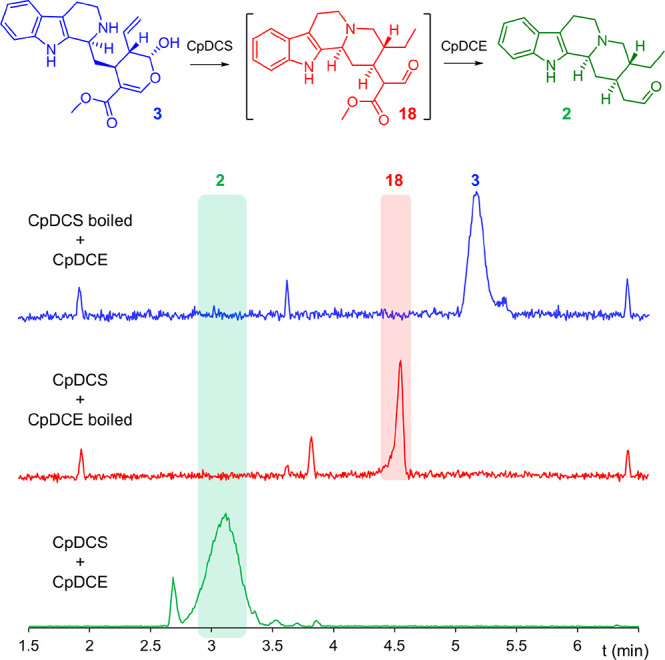
TIC traces of enzymatic assays featuring combinations of CpDCS and CpDCE.

We then considered the mechanism that the reductase CpDCS uses to catalyze the formation of **18** (Scheme 2a). After SGD-catalyzed deglycosylation, the open aglycone **3** forms corynantheine iminium **19**, which exists in equilibrium with dehydrogeissoschizine **20**. The reductase CpDCS might act on the iminium of **19** to yield the quinine intermediate corynantheine aldehyde **14** (Scheme 2a, route A). Alternatively, when the alkene is in conjugation with the iminium, as is the case with dehydrogeissoschizine **20**, the reductase can catalyze a 1,4-reduction of **20** followed by a 1,2 reduction of **22**, leading to the dihydro-series (Scheme 2a, route B). This consideration opens the possibility that the cell environment of *Cinchona* might regulate the flux toward quinine or the dihydro-series by tuning the equilibrium of dehydrogeissoschizine and corynantheine iminium **19**, thereby enhancing the variety of quinine-like scaffolds. Surprisingly, we never observed evidence of formation of corynantheine aldehyde **14** (*m*/*z* 353.19) or corynantheal **13** (*m*/*z* 295.18) with any of the 16 MDH candidates, even under limiting concentrations of NADPH cofactor. Either an as-yet unidentified reductase is responsible for the formation of corynantheine aldehyde **14** or the conditions that were used for our *in vitro* assays favor the formation of the dehydrogeissoschizine isomer **20** (Scheme 2); however, because CpDCS catalyzes a 1,2-iminium reduction in the formation of dihydrocorynantheine aldehyde **18**, it seems reasonable that this enzyme could also catalyze the 1,2-reduction of **19** to yield **14** (Scheme 2a). De-esterification of **18** by CpDCE then leads to a carboxylic acid that could spontaneously decarboxylate (Scheme 2b).

**Scheme 2:**
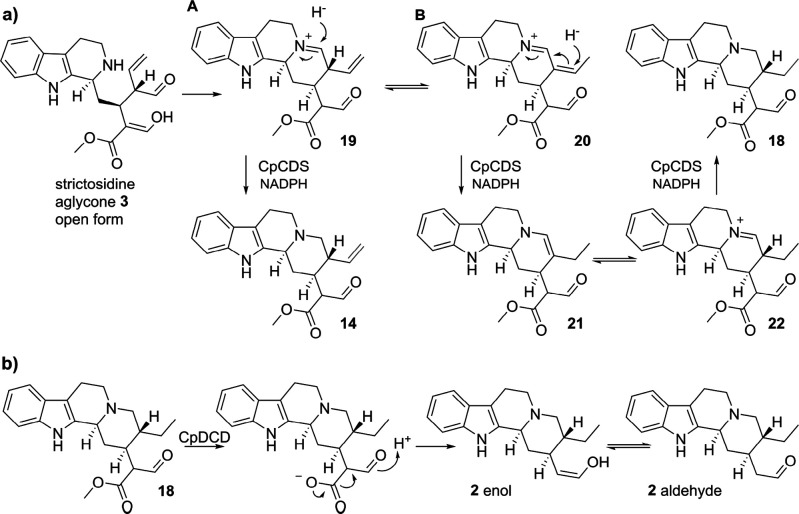
Formation of (Dihydro)corynantheal (a) Proposed mechanism of CpCDS, leading to the dihydro-series (route A) or quinine (route B). (b) Proposed action of CpDCE.

We next tested whether the orthologues of tabersonine 16-hydroxylase and 16-hydroxytabersonine *O*-methyltransferase were responsible for the late-stage methoxylation of the quinoline scaffold. An assay of 20 tabersonine-16-hydroxylase orthologues with cinchoninone **16/17**, cinchonine **6**, and cinchonidine **7** failed to yield any new product. The hydroxylation of the quinoline scaffold may not be catalyzed by an orthologue of tabersonine 16-hydroxylase, and more extensive candidate screening is required to identify the enzyme for this step. However, an orthologue of 16-hydroxytabersonine *O*-methyltransferase (CpOMT1) appeared to methylate a semisynthetically generated standard of 6′-hydroxycinchoninone **4** (Figure 3A, Figures S28–S31). The product was confirmed to be 6′-methoxycinchoninone **23** by comparison with an authentic chemical standard. This methyltransferase was also assayed with 6′-hydroxycinchonidine **25** and 6′-hydroxycinchonine **24** standards, but only a modest amount of **25** was converted to quinine **1** (Figure 3B,C). Quantification of substrate conversion with CpOMT1 in an endpoint assay with 100 μM substrate showed that only 17 ± 1% of 6′-hydroxycinchonidine **25** was consumed, whereas 80.4 ± 0.4% of 6′-hydroxycinchoninone **4** was consumed (Figures S32 and S33).

**Figure 3:**
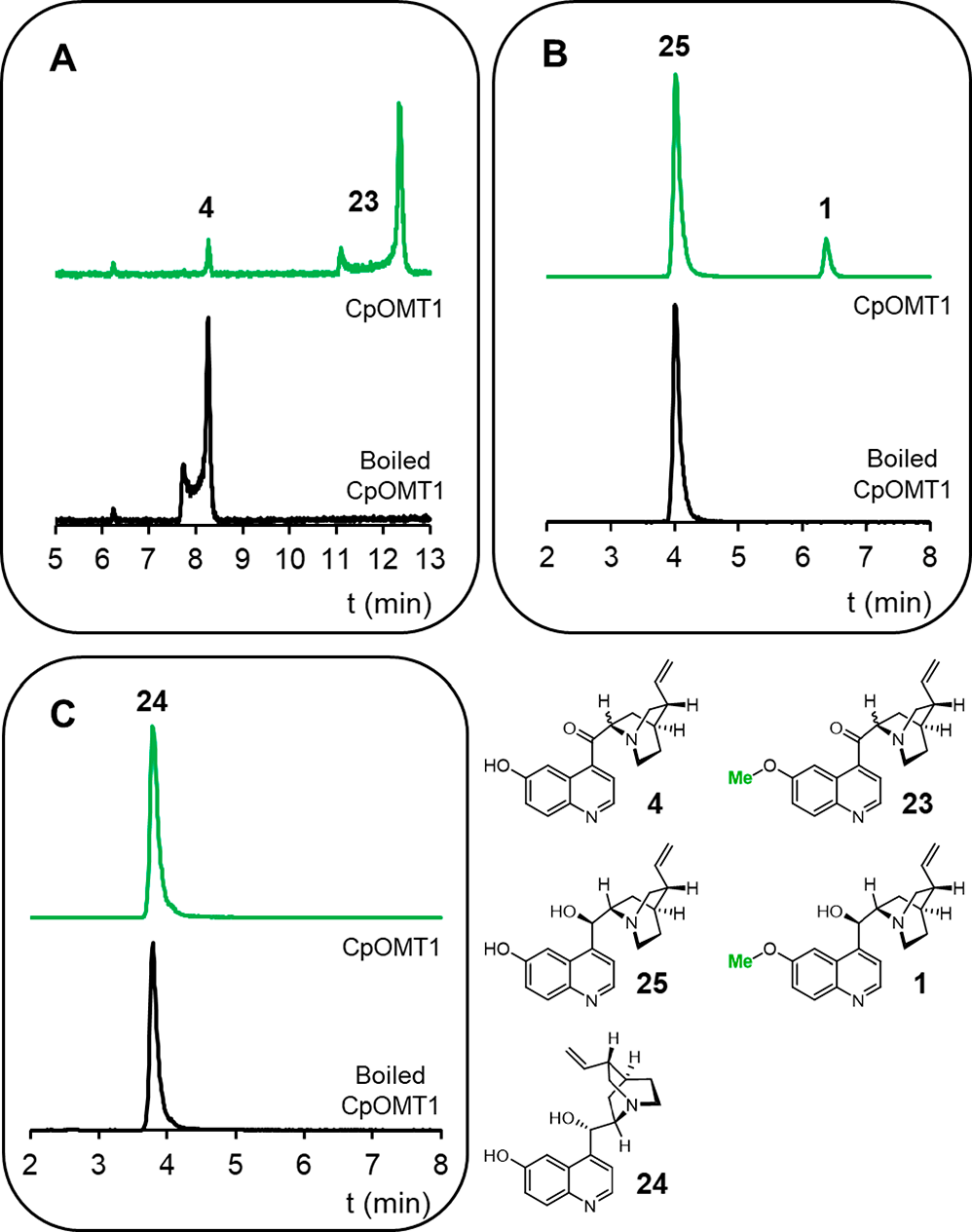
TIC traces of CpOMT1 assays using substrates **4** (A), **25** (B), and **24** (C) incubated with CpOMT1 for 12 h. Notice that the ketone analogs **4** are in an inseparable tautomeric equilibrium, as indicated by the two peaks in the chromatogram.

The higher relative activity of CpOMT1 for 6′-hydroxycinchoninone **4** suggests that the hydroxylation and methylation of the quinine scaffold on **16/17**, followed by NADPH-dependent keto-reduction, may be the preferred biosynthetic route *in planta* (Scheme 3, route A). However, we cannot exclude on the basis of the *in vitro* assays that the biosynthesis of quinine **1** (and quinidine **5**) might also go through hydroxylation and *O*-methylation of chinchonidine **7** (and chinchonine **6**), respectively (Scheme 3, route B).

**Scheme 3:**
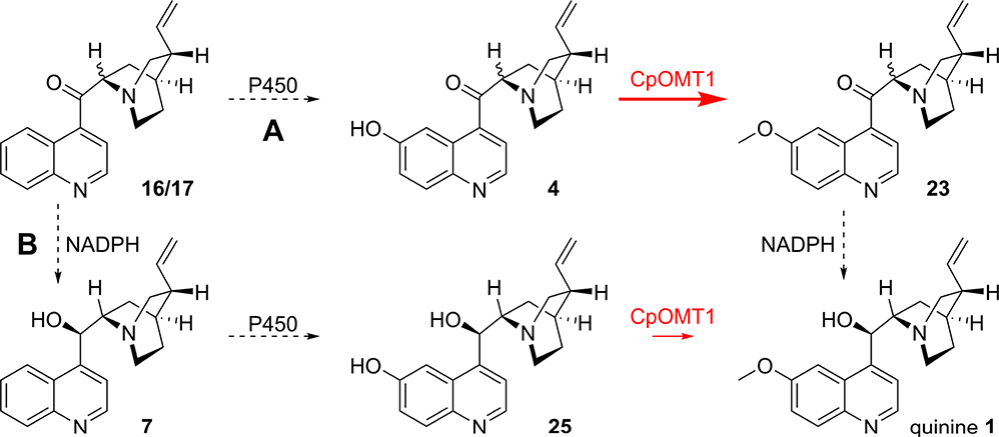
Proposed Order of Final Steps *In vitro* enzyme assays suggest that the substrate specificity of CpOMT1 favors route A over route B.

In conclusion, we identified a medium-chain alcohol dehydrogenase that reduces strictosidine aglycone **3** to yield dihydrocorynantheine aldehyde **18**. This chemical step also occurs in the biosynthesis of mitragynine, and we could identify an additional orthologue in the producer plant *Mitragyna speciosa*. We named these genes CpDCS (GenBank MW456554) and MsDCS (GenBank MW456555). Although we only observed the over-reduced product **18** that leads to the dihydro-series **8**–**11**, we hypothesize that control of the equilibrium of strictosidine aglycone **3** would yield corynantheine aldehyde **14**. We also discovered an orthologue of polyneuridine aldehyde esterase from *Rauwolfia serpentina*, CpDCE (GenBank MW456556) that acts on **18**, leading to demethylation, decarboxylation, and the formation of dihydrocorynantheal **2**. Finally, the discovery of CpOMT1 (GenBank MW456557) that acts preferentially on 6′-hydroxycinchoninone **4** rather than on the keto-reduced 6′-hydroxycinchonine **24** and 6′-hydroxycinchonidine **25** suggests that hydroxylation and methylation of the quinine scaffold occur on cinchoninone **16/17** followed by keto-reduction. The discovery of the enzymes that catalyze these three steps sets the stage for the elucidation of the remaining enzymes involved in quinine biosynthesis.

## Supporting Information Available

Detailed biochemical, molecular biology, metabolomic and synthetic procedures, enzyme assays, compound characterization, NMR spectra, additional experimental procedures, and spectroscopic data for all new compounds (PDF)

## Notes

Transcriptomic data is uploaded at Data Dryad (https://doi.org/10.5061/dryad.z34tmpgcc).
