# Supplementary material for: Early and Late Steps of Quinine Biosynthesis
Source: Org Lett. 2021 Feb 24;23(5):1793–7. doi: 10.1021/acs.orglett.1c00206 (PMC7944568; doi:10.1021/acs.orglett.1c00206)
Supplement: Supplementary file 1 — ol1c00206_si_001.pdf [file ol1c00206_si_001.pdf]

Supporting Information

Early and late steps of quinine biosynthesis

Francesco Trenti, Kotaro Yamamoto, Benke Hong, Christian Paetz, Yoko Nakamura, Sarah E. O'Connor

Department of Natural Product Biosynthesis, Max Planck Institute for Chemical Ecology, Hans-Knöll-Straße 8, 07745 Jena, Germany

Contents

Chemicals and molecular biology kits……………………..…………………………………………………………2
RNA purification and sequencing…………………….……………………………………………………………2
Mass spectrometers parameters………………………………………………………………………………………3
Untargeted UPLC/MS analysis……………………………………………………..…………………………………3
Targeted UPLC/MS analysis…………………………………………………………………………………………3
C. pubescens metabolite analysis…………………………………………..…………………………………………3
Candidate gene cloning…………………………………………………………………..……………………………3
Protein expression and purification…………………………………………………..………………………………4
E. coli soluBL21 expression and purification…………………………………………..……………………………4
S. cerevisiae expression and microsome preparation…………………….……………….…………………………4
Enzymatic assays…………………………………………………………………………………………………….5
MDH and esterase assays…………………………………………………………………..…………………………5
Cytochrome P450 assays…………………………………………………………………..…………………………5
Yeast feeding assays……………………………………………………………………………………………….…5
In vitro microsome assays………………………………………………………………………………….…………5
OMT enzyme assays……………………………………………………………………………………….…………5
CpOMT1 conversion rate……………………………………………………………………………….……………5
Dihydrocorynantheal purification…………………………………………………………………….………………6
NMR analysis……………………………………………………………………………………..……..………..………6
Synthesis of Quinine Analogs…………………………………………………………..…………..……………………7
Cinchoninone **16** and Cinchonidone **17**…………………………………………………………………………………7
6′-Hydroxycinchonidine **25**…………………………………………………………………………...…………7
6′-Hydroxycinchonine **24**………………………………………………………………………………….…………8
6′-Methoxycinchonidone **26** and 6′-Methoxycinchoninone **23**……………………...………………………………8
6′-Hydroxycinchoninone **4**………………………………………………………………………...……………………8
Supplementary Figures and Tables……………………………………………………………….……………………10
References……...…32

Materials

Chemicals and molecular biology kits

All solvents used for extractions, chemical synthesis and preparative HPLC were of HPLC grade, whilst solvents for UPLC/MS analysis were of MS grade. All solvents were purchased from Fisher Scientific. Quinine **1**, quinidine **5**, cinchonine **6** and cinchonidine **7** standards were purchased from Sigma-Aldrich. Notably all these compounds come with a contamination of their respective dehydro-forms as reported in their description sheet provided by Sigma-Aldrich. Strictosidine **12** was prepared as previously described.[1] Synthesis of cinchona alkaloids that are not commercially available is described below.

RNA extraction reagents, CTAB (Hexadecyltrimethylammonium bromide); PVP40 (Polyvinylpyrrolidone); Chloroform – isoamyl alcohol mixture (24:1); β-Mercaptoethanol; Lithium chloride (LiCl); spermidine trihydrochloride; SDS (Sodium dodecyl sulfate); diethylpyrocarbonate; and RNaseZap were purchased from Sigma-Adlrich. Ethanol absolute; EDTA pH 8; NaCl; Ultra Pure Tris-HCl buffer 1 M pH 8.0; and water sterile for RNA work were purchased from Fisher Scientific. Carbenicillin was purchased from Formedium.

All gene and fragment amplifications were performed using Platinum Superfi polymerase (Thermo Fisher) whilst colony PCR reactions were performed using Phire II master mix (Thermo Fisher). All primers were purchased from Sigma-Aldrich. PCR product purifications were performed using the Zymo Research PCR clean-up kit. Plasmids purifications were performed using Promega Wizard minipreps. cDNA was prepared using Superscript IV VILO master mix and Turbo DNAse (Thermo Fisher). All restriction enzymes were purchased from NEB.

Methods

RNA purification and sequencing

Plant material was harvested from roots and stems, as well as from young leaves. Total RNA was extracted using the CTAB protocol, which is an optimized procedure for extracting total RNA from tissues containing elevated levels of polysaccharides and polyphenolics compounds.[2, 3] All surfaces, gloves and equipment were cleaned by wiping with RNaseZap. Extraction buffer EB (CTAB 2% w/v; PVP40 2% w/v; Tris-HCl pH 8.0 100 mM; EDTA pH 8.0 25 mM; NaCl 2 M) (10 mL) was heated to 65 ºC and then 200 µL of β-mercaptoethanol and 0.05% spermidine trihydrochloride were added. Frozen ground tissue (100 mg) were extracted with 800 µL of warm EB and vortexed. Samples were incubated at 65 ºC for 10 minutes and vortexed 8 times vigorously during the incubation. Cooling at room temperature for 2 minutes followed. A solution of 24:1 chloroform:isoamyl alcohol (600 µL) was added and samples vortexed vigorously for 2 minutes. Samples were centrifuged at room temperature 12,000 *g* for 20 minutes, at which point the phases were clearly separated. The supernatant (~550 µL of viscous aqueous phase) was carefully transferred to a new 1.5 mL tube, and was re-extracted with ~550 µL of 24:1 chloroform:isoamyl alcohol solution and vortexed for 1 minute. Centrifugation and supernatant collection ensued. To precipitate nucleic acids, 0.25 volumes (~135 µL) of LiCl 10 M was added to the combined viscous aqueous phases. Samples were mixed by inverting the tubes and incubated overnight at 4 ºC. The next day, RNA was pelleted by centrifuging the samples at 14,000 *g* for 40 minutes at 4 ºC. The supernatant was carefully discarded using a pipette. The pellets were resuspended in 500 µL SSTE buffer (NaCl 1 M; SDS 0.5%; Tris-HCl pH 8.0 10 mM; EDTA pH 8.0 1 mM) and vortexed for ~10 seconds. The samples were extracted with equal volume (500 µL) of 24:1 chloroform:isoamyl alcohol and vortexed. Centrifugation at 14,000 *g* for 30 minutes at 4 ºC followed until phase separation. The viscous aqueous phase (~500 µL) was carefully recovered to a fresh 1.5 mL tube. Ice-cold absolute ethanol (100%) (2 volumes, ~ 1000 µL) was added and mixed well by vortexing for 10 seconds. RNA was precipitated at -80 ºC for 90 minutes. After incubation, samples were centrifuged at 14,000 *g* for 30 minutes at 4 ºC, and the supernatant carefully removed. The pellets were washed with 1 mL of 75% ice cold ethanol and mixed well by vortexing for 10 seconds. The samples were centrifuged at 14,000 *g* for 30 minutes at 4 ºC, and supernatant was discarded. The pellets were completely dried at room temperature for ~30 minutes under sterile clean bench. The pellet containing total RNA was dissolved in 30 µL of water (0.1% diethylpyrocarbonate; DNase, RNase free). Quantification and quality control were performed spectrophotometrically by analyzing absorption ratios: A_{260}/A_{240} and A_{260}/A_{280}. Absorbance ratios detect polysaccharide/polyphenolic contaminants and protein contaminates respectively. Typical absorbance ratio for good quality RNA are A_{260}/A_{240} ~1.9 and A_{260}/A_{280} ~2.1. Samples were stored at -80 ºC.

Each sample was prepared in 5 biological replicates (Cp1-5). Samples with RNA concentration ranging from 100-500 ng/µL and acceptable quality (A_{260}/A_{240} and A_{260}/A_{280} ~2) were sent to BGI-seq for sequencing, assembly and annotation.

Mass spectrometers parameters

Untargeted UPLC/MS analysis

Samples were analyzed using Bruker Impact II high resolution Quadrupole Time-Of-Flight mass spectrometer (Q-TOF) coupled with ELUTE LC system.

Method 1

Ionization was performed via pneumatic-assisted electrospray ionization in positive mode (ESI+) with a capillary voltage of 4500 V and an end plate offset of 500 V; a nebulizer pressure of 2.5 bar was used, with nitrogen at 250°C and a flow of 11 L/min as the drying gas. Acquisition was done at 12 Hz following a mass range from 20 to 1000 *m/z*, with data-dependent MS/MS and an active exclusion window of 0.2 min, a reconsideration threshold of 1.8-fold change. Fragmentation was triggered on an absolute threshold of 400 and acquired on the most intense peaks using a target intensity of 20000 counts, with MS/MS spectra acquisition between 12 and 20 Hz, and limited to a total cycle time range of 0.5 s. Collision energy was determined automatically by the software depending on *m/z* value. At the beginning of each run, an injection of a sodium formate-isopropanol solution was performed and the *m/z* values were re-calibrated using the expected cluster ion *m/z* values. Samples were run in at a flowrate of 0.6 mL/min in a formic acid 0.1% - acetonitrile (plus 0.1% formic acid) gradient linearly increasing from 5% to 20% acetonitrile in 15 minute using analytical Phenomenex Kinetex column (2.6 µm; XB-C18; 100 Å; column temperature 40°C). 2 µL of samples were injected.

Method 2

As Method 1, with exception of capillary voltage set at 3500 V and mass range from 800 to 1000 *m/z*. Samples were run in at a flowrate of 0.6 mL/min in a formic acid 0.1% - acetonitrile (plus 0.1% formic acid) gradient linearly increasing from 10% to 30% acetonitrile in 6 minutes using analytical Phenomenex Kinetex column (2.6 µm; XB-C18; 100 Å; column temperature 40°C). 2 µL of samples were injected.

Targeted UPLC/MS analysis

Bruker EVOQ Elite triple quadrupole MS system coupled with ELUTE LC system was used in multiple reaction monitoring (MRM) mode. Ionization was performed via heated electrospray ionization (HESI) in positive mode with a capillary voltage of 3500 V; cone temperature of 350 ºC; cone gas flow set at 20; heated probe temperature of 450 ºC; nebulizer gas flow set at 50; and probe gas flow set at 45. The source conditions were optimized using authentic standards of quinine **1**, 6'-methoxycinchoninone **23**, 6'-hydroxycinchonidine **25**, and 6'-hydroxycinchonidone **4**. Compounds with ions quantifiers' and qualifiers' collision energies are listed in Table S1. Note that for every sample, the doubly charged *m/z* values were used as parental ions. Samples were run in a gradient 0.1% formic acid-acetonitrile (0.1% formic acid) in a two-step gradient of 5 minutes total and 0.6 mL/min: (first step) linear gradient 10%-17% acetonitrile for 3 minutes and (second step) linear gradient 17%-40% acetonitrile for 2 minutes using analytical Phenomenex Kinetex column (2.6 µm; XB-C18; 100 Å; column temperature 40°C). 2 µL of samples were injected.

C. pubescens metabolites analysis

The same plant material used for RNA analysis was also subjected to metabolomic analysis. Plant material was snap-frozen with liquid nitrogen, ground with mortar and pestle and extracted with MeOH. The volume of MeOH was normalized on fresh tissue weight (100:1 = mg:mL). Plant material was centrifuged at 16000 *g* for 10 minutes and the supernatant filtered with Fisher polytetrafluoroethylene (PTFE) syringe filters (0.2 µm). The resulting methanolic extracts were diluted 1:10 in methanol and analyzed by untargeted UPLC/MS (Method 1). Extracted ion chromatograms are shown for each organ with standards (Figure S1).

Candidate gene cloning

The full-length sequences of the genes of interest were amplified from *C. pubescens* cDNA using the primers listed in Table S2. Genes annotated as medium-chain alcohol dehydrogenases (MDHs), esterases or *O*-methyl transferases (OMT) were cloned into either pOPINF or pOPINM vectors between BamHI and KpnI restrictions sites.[4] Cytochrome P450 genes were cloned into pESC-leu2d::AaCPR vector suitable for yeast protein expression (Twist Bioscience). Note that pESC

vector encodes a copy of the *Artemisia annua* Cytochrome P450 Reductase necessary for P450 activity. The PCR products were purified from agarose gel and ligated into the suitable vector using the In-Fusion kit (Clontech Takara). Infusion reactions were transformed into competent *E. coli* Stellar cells (Clontech Takara). Recombinant colonies were selected on LB agar plates supplemented with carbenicillin (100 µg/mL). Positive clones were identified by colony PCR using sequencing primers designed for the specific vector used (Table S2). Plasmids were isolated from positive colonies grown overnight. Identities of the inserted sequences were confirmed by Sanger sequencing.

Protein expression and purification

Soluble medium-chain alcohol dehydrogenases, esterases and *O*-methyltransferases were expressed in *E. coli* soluBL21, while membrane-bound cytochromes P450 were expressed in *S. cerevisiae* PEP4KO.

E. coli soluBL21 expression and purification

E. coli soluBL21 were transformed with plasmids carrying the gene of interest by heat shock (ice, 20 minutes; 42 ºC, 45 seconds). Transformed cells were selected by growing overnight at 37 ºC on LB Agar plus carbenicillin 100 µg/mL. A single positive colony was grown in a pre-culture of 10 mL LB medium and incubated overnight at 37 ºC with shaking. The following day, 1 mL of this overnight culture was used to inoculate 100 mL of LB medium and grown at 37 ºC until reaching of OD_{600} = 0.6 - 1. At this cell density, cultures were cooled on ice and induced with IPTG 300 µM. Cultures were incubated overnight at 18 ºC shaking.

All steps for protein purification were performed on ice with cooled buffers and centrifuges. Bacterial cells were pelleted and resuspended in 15 mL A1 buffer (50 mM Tris-HCl pH 8; 50 mM glycine; 500 mM NaCl; 5% glycerol; 20 mM imidazole) with addition of EDTA-free protease inhibitors cocktail (Sigma-Aldrich), then incubated on ice for 30 minutes. Cell disruption was performed by sonication (20 KHz; Amplitude 40%; 2 minutes) and cell membranes were pelleted by centrifugation at 35,000 *g* for 30 minutes. The supernatant was filtered through syringe glass-filter (Sartorius) and 200 µL of NiNTA beads were added. NiNTA beads were washed three times with 1 mL A1 buffer prior to addition. The supernatant plus NiNTA beads was incubated shaking at 4 ºC for 30 minutes. After incubation, NiNTA beads were pelleted and washed three times with buffer A1. Elution of the proteins was performed twice by resuspending the beads in 600 µL Buffer B (50 mM Tris-HCl pH 8; 50 mM glycine; 500 mM NaCl; 5% glycerol; 500 mM imidazole). Dialysis and buffer exchange was performed using Buffer A4 (20 mM HEPES pH 7.5; 150 mM NaCl) in centrifugal concentrators with size exclusion of 10 or 30 KDa depending on the protein size. Proteins were aliquoted in 30 µL, snap-frozen and stored at -80 ºC.

S. cerevisiae expression and microsomes preparation

Yeast *S. cerevisiae* PEP4KO was transformed with pESC vector carrying the gene of interest, using the LiAc/SS-DNA/PEG protocol.[5] Yeast was inoculated in 10 mL YPAD medium (yeast extract 1%; peptone 2%; glucose 2%; adenine sulfate dihydrate 40 mg/L) and incubated at 30 ºC shaking overnight. The next day, the 10 mL culture was diluted 1:10 in 100 mL YPAD and incubated at the same conditions for 4-5 hours. After incubation, cells were pelleted by centrifugation (top speed, 5 minutes) and washed twice with sterile water. Cells were resuspended in transformation mix (for each transformation: 120 µL 50% PEG; 18 µL 1 M LiAc; 25 µL 2 mg/mL boiled carrier salmon sperm DNA) and aliquoted in 1.5 mL tubes. For each transformation 100-500 ng of plasmid DNA was added and mixed by pipetting. Transformation tubes were incubated at 42 ºC in water bath for 30-40 minutes. After incubation, tubes were placed on ice for 1-3 minutes, then cells were pelleted and supernatant carefully removed. The cell pellet was resuspended in 150 µL sterile water and plated on Synthetic Complete SC-Leu Agar (Difco Yeast Nitrogen base without amino acids 0.335%; Sigma Yeast Synthetic Drop-Out medium supplemented without Leucine 0.196%; agar 2%; mono + dibasic sodium phosphate pH 7.4 5%) with addition of glucose 2%. Positive colonies appeared after 3-5 days of incubation at 30 ºC.

A single positive colony was grown in 10 mL SC-Leu + 2% glucose at 30 ºC overnight. The next day P450 expression was induced by inoculating all 10 mL into 100 mL of SC-Leu + 2% galactose and induced at 30 ºC for 24-48 hours. After protein induction microsomes were prepared by resuspending yeast cells into 30 mL TEK Buffer (50 mM Tris-HCl; 1 mM EDTA; 0.1 M KCl pH 7.4) and incubated for 5 minutes at room temperature. Cells were pelleted (4000 *g*; 10 minutes; 4 ºC) and resuspended in 20 mL TES-B buffer (50 mM Tris-HCl; 1 mM EDTA; 0.6 M sorbitol pH 7.4). Cells were lysed using cell disruptor (30 KPa). Lysate was centrifuged at 11000 *g* for 20 minutes at 4 ºC to precipitate intact cells, organelles and membranes. The supernatant was centrifuged in an ultra-centrifuge at 100000 *g* for 1 hour at 4 ºC. The

resulting membrane pellet was resuspended in 150-300 µL of TEG buffer (50 mM Tris-HCl; 1 mM EDTA; 20% glycerol; pH 7.4) and gently mixed by pipetting. Microsomes were aliquoted, snap-frozen and kept at -80 ºC.

Enzymatic assays

Enzyme candidates were tested as purified proteins in case of soluble MDH, esterases and OMTs. Cytochromes P450s were used as microsomal preparations or, alternatively, were tested by directly culturing the appropriate yeast cultures with the substrate present in the media at 100 µM concentration. For negative control experiments, soluble protein samples were replaced with boiled protein. For membrane bound P450s, the controls consisted of microsomes prepared from yeast transformed with pESC-leu2d::AaCPR empty vector, or in case of direct substrate feeding, yeast transformed with pESC-leu2d::AaCPR empty vector. After incubation, the reactions were quenched by addition of 1 volume of methanol, filtered through 0.22-µm nylon Spin-X centrifuge filters (Corning) and analyzed by untargeted UPLC/MS.

MDH and esterase assays.

MDH enzymes were tested in the presence of strictosidine **12** and *C. roseus* strictosidine glucosidase (CrSGD) to generate the strictosidine aglycone **3** substrate. Strictosidine aglycone **3** is highly reactive and thus cannot be isolated for these assays. Reaction mixtures consisted of MDH (1 µM) and CrSGD (1 µM) in a total volume of 50 µL HEPES pH 7.5 (50 mM); strictosidine **12** (100 µM); NADPH (100 µM). Reactions were incubated at 30 ºC for 1 hour. After incubation, the reactions were quenched by addition of 1 volume of methanol, filtered through 0.22-µm nylon Spin-X centrifuge filters (Corning) and analyzed by untargeted UPLC/MS (Method 2).

Since the dihydrocorynantheine aldehyde **18** product could not be successfully isolated, the activity of the esterases were assayed in the presence of CrSGD, the appropriate MDH (CpDCS) and strictosidine **12**, using the same conditions described above, with the addition of the esterase protein to a final concentration of 1 µM.

Cytochrome P450 assays

Yeast feeding assays

Transformed yeast was grown in 10 mL SC-Leu + 2% glucose media overnight. The following day, 100 µL of culture was aliquoted and cells pelleted. To the cell pellet, 100 µL of SC-Leu + 2% galactose + the substrate (final concentration of 100 µM) was added. Substrates tested were: cinchonine **6**; cinchonidine **7**; and the cinchoninone **16** - cinchonidone **17** mix. The inducing medium was adjusted to pH 8 with Tris-base. Cultures were incubated with shaking for 24-48 hours at 30 ºC. After incubation cells were pelleted and the supernatant was passed through a SPE column (Waters). Samples were analyzed by untargeted UPLC/MS (Method 1).

In vitro microsome assays

Microsomes were tested with substrates cinchonidine **7**; cinchonine **6**; cinchoninone **16** in excess of NADPH (1 mM). A total of 10 µL of microsomes was used in a final volume of 100 µL. The reactions were carried in HEPES pH 7.5 50 mM and incubated at 30 ºC overnight. The next day, one volume of methanol was added, and microsomes pelleted by centrifugation. The supernatant was analyzed by untargeted UPLC/MS (Method 1).

OMT enzyme assays

OMT enzymes were tested using as substrates 6′-hydroxycinchonine **24**; 6′-hydroxycinchonidine **25**; and 6′-hydroxycinchoninone **4**, which exist in an inseparable tautomeric equilibrium. Assays were performed in 50 µL volume of Tris-base pH 8 50 mM; substrate concentration varied between 50 and 100 µM; SAM (200 µM) and ascorbate (200 µM) were used as cofactors; and the enzymes were diluted to a final concentration of 1-5 µM. Negative controls consisted of boiled OMT in the reaction mixture. Reactions were incubated at 30 ºC for 1 to 12 hours. After incubation, the reactions were quenched by addition of 1 volume of methanol, filtered through 0.22-µm nylon Spin-X centrifuge filters (Corning) and analyzed by untargeted UPLC/QTOF (Method 1) (Figures S3).

CpOMT1 conversion rate

The conversion rate for the CpOMT1, was measured. CpOMT1 (1 µM) was incubated with 6'-hydroxycinchoninone **4** (100 µM) or 6'-hydroxycinchonidine **25** (100 µM) in the presence of SAM (200 µM) and ascorbate (200 µM) in Tris-Base pH 8.5 (50 mM) at 30 ºC for 12 hours. All reactions were performed in triplicate. After incubation, the reactions were quenched by addition of 1 volume of methanol, filtered through 0.22-µm nylon Spin-X centrifuge filters (Corning) and analyzed by untargeted UPLC/MS (Method 1) (Figure S3).

To estimate the end point conversion the samples were measured by targeted UPLC/MS. Calibration curves were calculated for all substrates (6'-hydroxycinchonidine **25** and 6'-hydroxycinchoninone **4**) and products (quinine **1** and 6'-methoxycinchonidine **18**) by measuring triplicates of serial dilutions ranging from 12 nM to 800 nM and plotting each compound concentration against the peak integrals (Figure S4). Peak area was calculated for the substrates and products of CpOMT1 reactions diluted to fall within the linear range and their concentration was calculated using the calibration plot equation. Conversion was calculated as percentage of end point product concentration.

Dihydrocorynantheal purification

Dihydrocorynantheal **2** was produced by enzymatic conversion of strictosidine **12** using purified CrSGD, CpDCS, and CpDCE enzymes. The reaction mix consisted of 1 mL strictosidine **12** (300 µM); HEPES pH 7.5 50 mM; NADPH 300 µM; and the three enzymes, each at 1 µM. The reaction was incubated at 30 ºC for 1 hour. A total of 10 reactions was run in parallel. After incubation, the samples were concentrated by SPE columns (Waters) and injected on Agilent HPLC system 1260 Infinity II and fractionated by Agilent 1290 Infinity II system. A semipreparative column was used (Phenomenex; Kinex 5µm XB-C18 100 Å; 250 x 10.0 mm) and a linear gradient 10%-30% acetonitrile in formic acid 0.1% in 35 minutes. The compound of interest was detected by UV absorption at wavelengths: 240 nm; 254 nm; and 290 nm. All fractions containing dihydrocorynantheal **2** were combined and dried by rotavapor. The product was then collected in 2 mL methanol, transferred in a glass vial and dried under argon at room temperature. Approximately 1 mg of dihydrocorynantheal **2** was recovered, and then subjected to NMR analysis (Figures S5-S23, Table S3). The enzymatic product was also analyzed by high resolution untargeted UPLC/MS (Method 2) and its molecular formula calculated using the function SmartFormula of the Compass DataAnalysis software (Bruker Compass HyStar 5.1 v.5.1.8.1). Tolerance was set at 2 ppm and charge state to +1. The analysis showed measured *m/z* 297.1959 with an error of 0.7 ppm, resulting in a calculated sum formula of $C_{19}H_{24}N_2O$.

NMR analysis

NMR spectra for dihydrocorynantheal **2** were measured on a 700 MHz Bruker Advance III HD spectrometer (Bruker Biospin GmbH, Rheinstetten, Germany). MeCN-d_3 was used as a solvent. NMR spectra were referenced to the residual solvent signals at δ_H 1.96 and δ_C 118.26 ppm. For spectrometer control and data processing Bruker TopSpin ver. 3.6.1 was used (Figures S5-S23, Table S3).

For quinine analogs synthetic standards, NMR spectra were measured on a 400 MHz Bruker Advance III HD spectrometer (Bruker Biospin GmbH, Rheinstetten, Germany). $CDCl_3$ was used as a solvent. NMR spectra were referenced to the residual solvent signals at δ_H 7.26 and δ_C 77.0 ppm. For spectrometer control and data processing Bruker TopSpin ver. 3.6.1 was used (Figures S24-S27).

Synthesis of Quinine Analogs

Cinchoninone **16** and Cinchonidone **17**

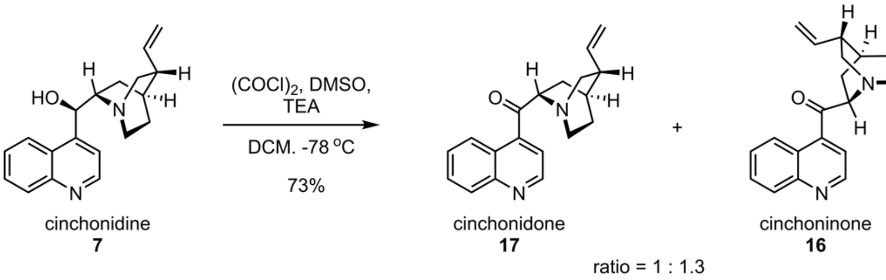

To a stirred solution of DMSO (19 µL, 0.267 mmol) in anhydrous DCM (1 mL) was added oxalyl chloride (9.2 µL, 0.1079 mmol) drowpwise at -78 ºC. After 30 min, cinchonidine **7** (15 mg, 0.0514 mmol) in anhydrous DCM (1 mL) was added dropwise at -78 ºC and the resulting mixture was stirred for 1 h at the same temperature. Then Et_3N (64 µL, 0.457 mmol) was added at -78 ºC and stirred for 30 min. The reaction was quenched by 2 mL brine extracted with DCM, dried over anhydrous Na_2SO_4 and concentrated in vacuum. The resulted residue was purified by column chromatography (DCM/MeOH = 20/1) to afford cinchonidone **17** and cinchoninone **16** (11 mg, 73%) as a mixture (ratio = 1:1.3). The spectroscopic data are in accordance with the literature values reported.[6, 7]

6´-Hydroxycinchonidine **25**

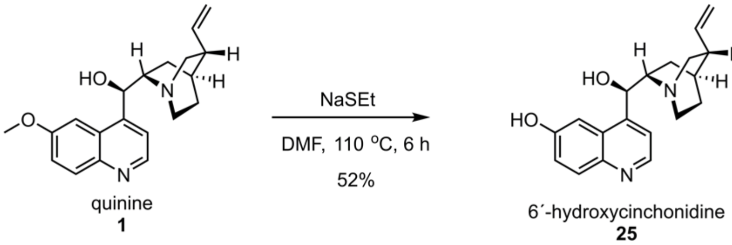

To a stirred solution of quinine **1** (20 mg, 0.0617 mmol) in anhydrous DMF (0.6 mL) was added sodium ethane sulfide (37 mg, 0.0.246 mmol) at room temperature. The resulting mixture was heated at 110 ºC for 6 hours. After cooling the reaction to room temperature, the reaction mixture was quenched with sat. NH_4Cl (1 mL), extracted with EtOAc, dried over anhydrous Na_2SO_4 and concentrated in vacuum. The resulted residue was purified by column chromatography (DCM/MeOH = 10/1) to afford 6´-hydroxycinchonidine **25** (10 mg, 52%). The spectroscopic data are in accordance with the literature values reported.[8]

6´-Hydroxycinchonine **24**

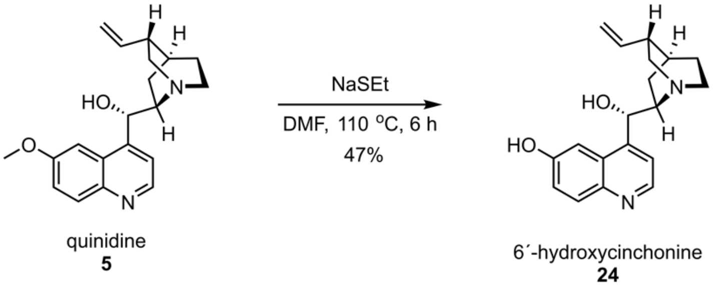

To a stirred solution of quinidine **5** (20 mg, 0.0617 mmol) in anhydrous DMF (0.6 mL) was added sodium ethane sulfide (37 mg, 0.0.246 mmol) at room temperature. The resulting mixture was heated at 110 ºC for 6 hours. After cooling the reaction to room temperature, the reaction mixture was quenched with sat. NH_4Cl (1 mL), extracted with EtOAc, dried over anhydrous Na_2SO_4 and concentrated in vacuum. The resulted residue was purified by column chromatography (DCM/MeOH = 10/1) to afford 6′-hydroxycinchonine **24** (9 mg, 47%). The spectroscopic data are in accordance with the literature values reported.[8]

6′-Methoxycinchonidone **26** and 6′-Methoxycinchoninone **23**

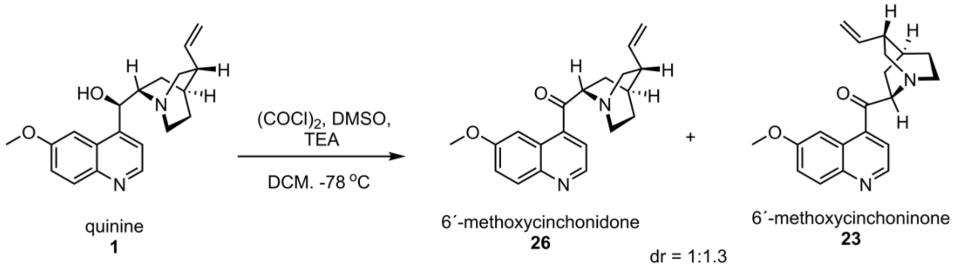

To a stirred solution of DMSO (38 µL, 0.534 mmol) in anhydrous DCM (2 mL) was added oxalyl chloride (18.4 µL, 0.216 mmol) drowpwise at -78 ºC. After 30 min, quinine **1** (34 mg, 0.103 mmol) in anhydrous DCM (2 mL) was added dropwise at -78 ºC and the resulting mixture was stirred for 1 h at the same temperature. Then Et_3N (128 µL, 0.914 mmol) was added at -78 ºC and stirred for 30 min. The reaction was quenched by 4 mL brine extracted with DCM, dried over anhydrous Na_2SO_4 and concentrated in vacuum. The resulted residue was purified by column chromatography (DCM/MeOH = 20/1) to afford a mixture of 6′-methoxycinchonidone **26** and 6′-methoxycinchoninone **23** (30 mg, 88%, ratio = 1:1.3). The spectroscopic data are in accordance with the literature values reported.[9]

6′-Hydroxycinchoninone **4**

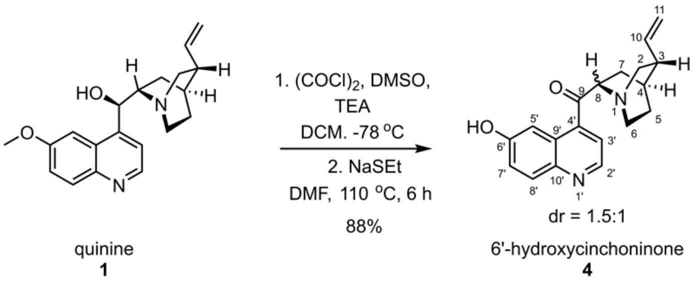

To a stirred solution of DMSO (19 µL, 0.267 mmol) in anhydrous DCM (1 mL) was added oxalyl chloride (9.2 µL, 0.1079 mmol) drowpwise at -78 ºC. After 30 min, quinine **1** (17 mg, 0.0514 mmol) in anhydrous DCM (1 mL) was added dropwise at -78 ºC and the resulting mixture was stirred for 1 h at the same temperature. Then Et_3N (64 µL, 0.457 mmol) was added at -78 ºC and stirred for 30 min. The reaction was quenched by 2 mL brine extracted with DCM, dried over anhydrous Na_2SO_4 and concentrated in vacuum. The resulted residue was used in next step directly.

To a stirred solution of above residue in anhydrous DMF (0.5 mL) was added sodium ethane sulfide (28 mg, 0.189 mmol) at room temperature. The resulting mixture was heated at 110 ºC for 6 hours. After cooling the reaction to room temperature, the reaction mixture was quenched with sat. NH_4Cl (1 mL), extracted with EtOAc, dried over anhydrous Na_2SO_4 and concentrated in vacuum. The resulted residue was purified by column chromatography (DCM/MeOH = 50/1 to 10/1) to afford a mixture of diastereomers (14 mg, 88%).

^{1}H NMR (400 MHz, $CDCl_3$, 1.5:1 mixture of diastereomers, * denotes major diastereomer) δ 8.76 (s, 1H, H2'), 8.75 (s, 1.5H, H2'*), 7.91 (d, *J* = 2.7 Hz, 1.5H, H5'*), 7.90 (d, *J* = 9.4 Hz, 1H, H8'), 7.86 (d, *J* = 9.1 Hz, 1.5H, H8'*), 7.76 (d, J = 2.6 Hz, 1H, H5'). 7.55 (d, J = 4.6 Hz, 1H, H3'*), 7.50 (d, J = 4.5 Hz, 1H, H3'), 7.26 (dd, *J* = 9.1, 2.6 Hz, 1H, H7'), 7.21 (dd, *J* = 9.1, 2.6 Hz, 1.5H, H7'*), 5.95 (ddd, *J* = 17.3, 10.5, 7.0 Hz, 1H, H10). 5.81 (ddd, J = 17.4, 10.3, 7.2 Hz, 1.5 H, H10*), 5.11-5.16 (m, 2H, H11), 5.07-4.99 (m, 3H, H11*), 4.57 (t, *J* = 9.6 Hz, 2H, H8*), 4.47 (t, *J* = 9.1 Hz, 1H, H8), 3.34-3.21 (m, 2.5H, H2, H2*), 3.20 – 3.0 (m, 6.5H, H2, H2*, H6, H6*), 2.84-2.81 (m, 1H, H6), 2.47-2.31 (m, 2.5H, H3, H3*),

2.12 – 2.07 (m, 1.5H, H7*), 2.01-1.91(m, 3.5H, H7, H4, H4*), 1.90 – 1.70 (m, 5.5H, H7, H7*, H5*), 1.63 – 1.48 (m, 2H, H5);

^{13}C NMR (101 MHz, $CDCl_3$, 1.5:1 mixture of diastereomers, * denotes major diastereomer) δ 201.8 (C9), 200.9 (C9)*, 157.6 (C6')*, 157.4 (C6'), 146.0 (C2'), 145.8 (C2')*, 144.7 (C4')*, 144.6 (C4'), 140.8 (C10), 139.6 (C10'), 139.2 (C10)*, 138.4 (C10')*, 131.3 (C8'), 131.2 (C8')*, 126.0 (C9')*, 125.8 (C9'), 122.8 (C7'), 122.7 (C7')*, 120.2 (C3')*, 119.9 (C3'), 115.7 (C11)*, 115.2 (C11), 106.9 (C5')*, 106.6 (C5'), 62.5 (C8), 61.7 (C8)*, 54.7 (C2), 49.1 (C6)*, 48.3 (C2)*, 43.0 (C6), 39.3 (C3)*, 38.9 (C3), 27.7 (C4)*, 27.2 (C4), 26.9 (C5), 26.2 (C5)*, 23.4 (C7)*, 23.3 (C7).

Supplementary Figures and Tables

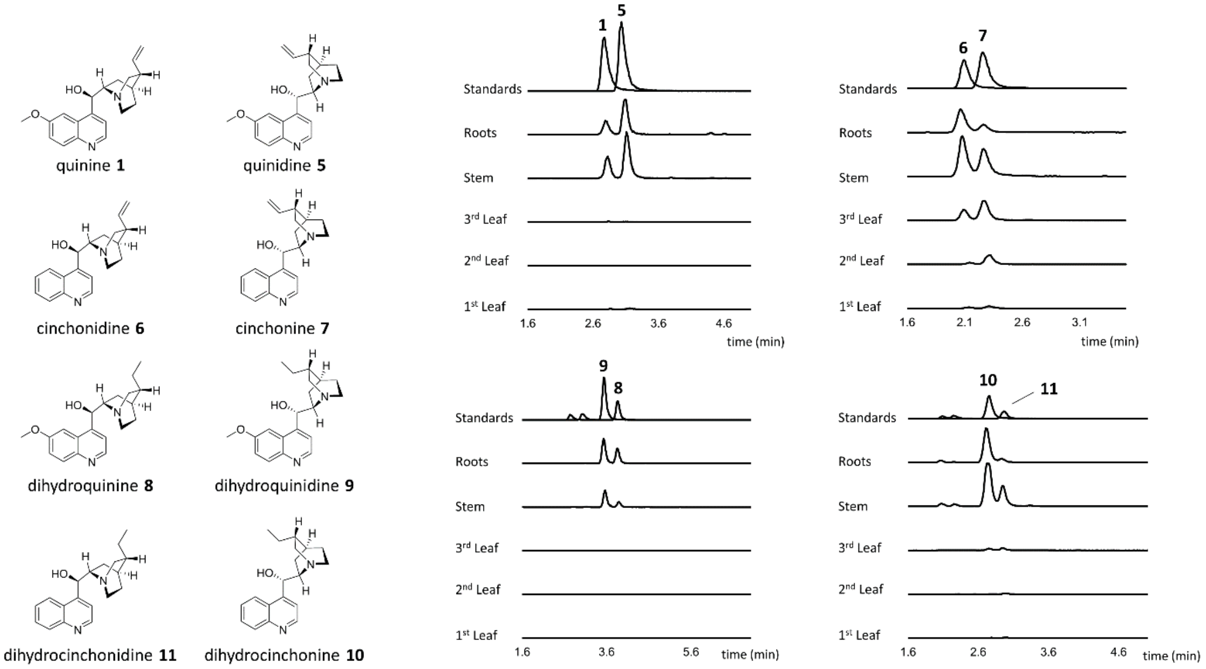

Figure S1: Extracted-ion chromatograms of *C. pubescens* leaves (1st; 2nd; and 3rd); stem; and roots. Plant extracts were compared with purchased quinine and analogs standards.

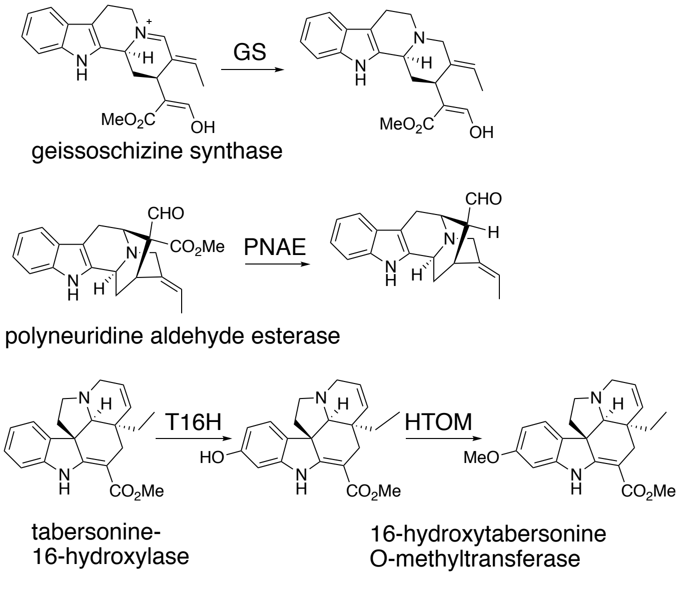

Figure S2: Enzymatic reactions of geissoschizine synthase, polyneuridine aldehyde esterase, tabersonine-16-hydroxylase and 16-hydroxytabersonine O-methyltransferase, which were used to search for gene candidates in *Cinchona*.

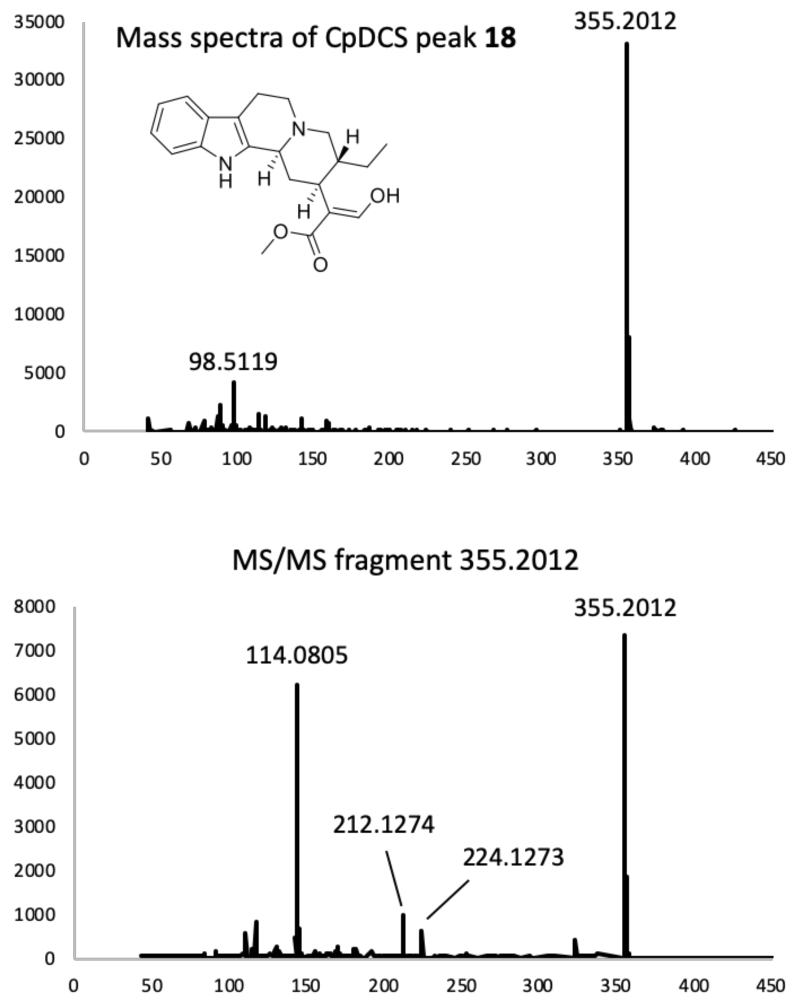

Figure S3: Mass spectra and MS/MS fragmentation of peak **18**.

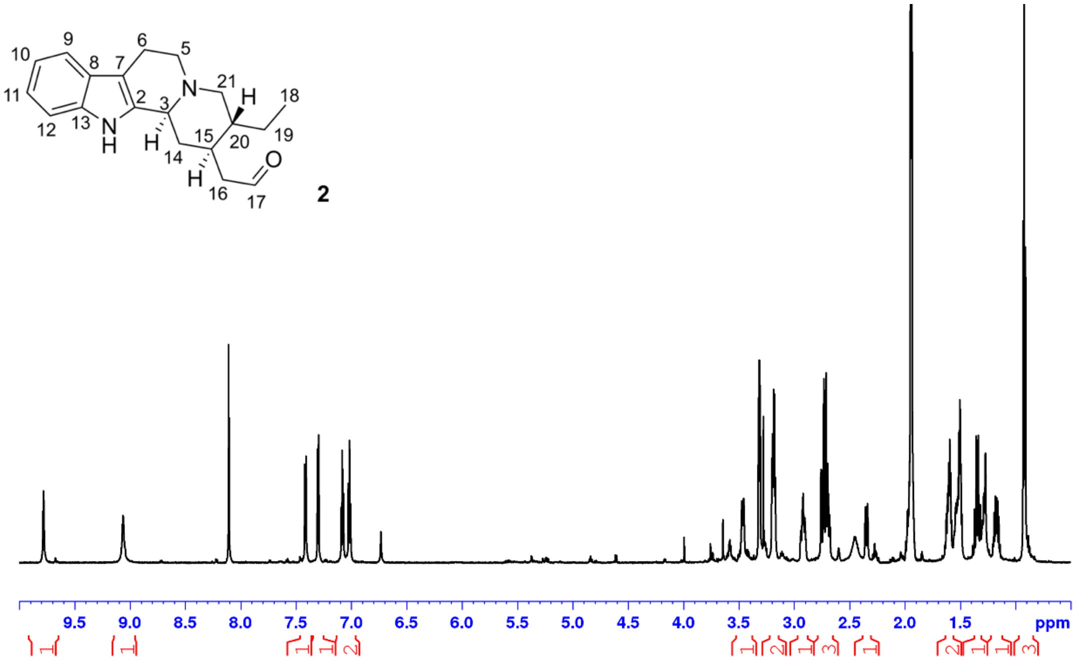

Figure S4: ^{1}H NMR of dihydrocorynantheal **2** with water suppression (zgpr), transmitter on 2.25 ppm, full range, in MeCN-d_3.

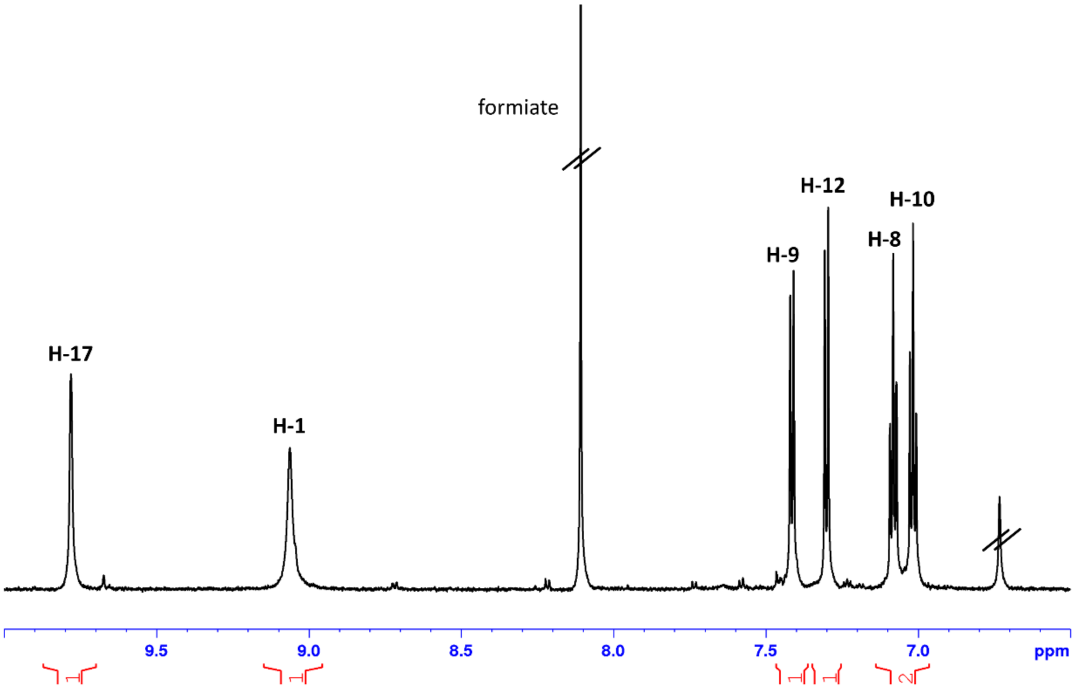

Figure S5: ^{1}H NMR of dihydrocorynantheal **2** with water suppression (zgpr), detail, aromatic range, in MeCN-d_3.

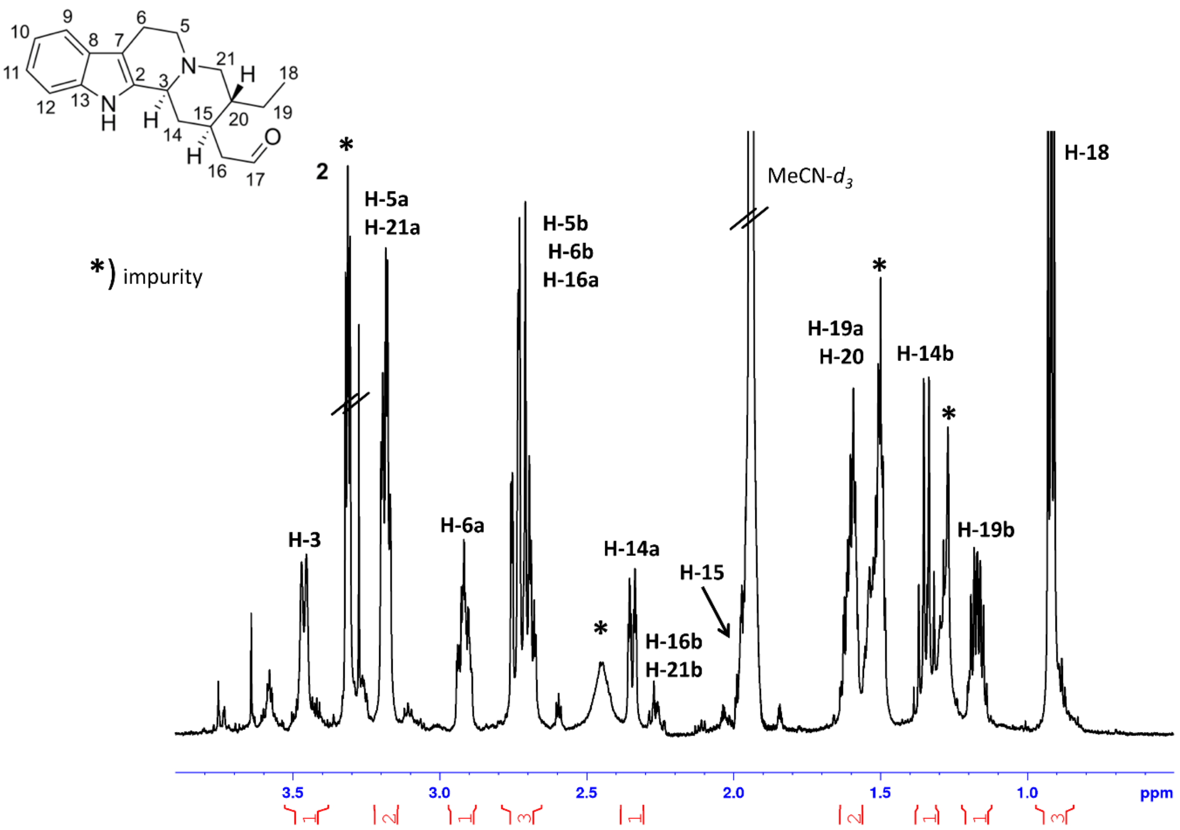

Figure S6: ^{1}H NMR of dihydrocorynantheal **2** with water suppression (zgpr), detail, aliphatic range. Intensity of H-16b/21b is attenuated, in MeCN-d_3.

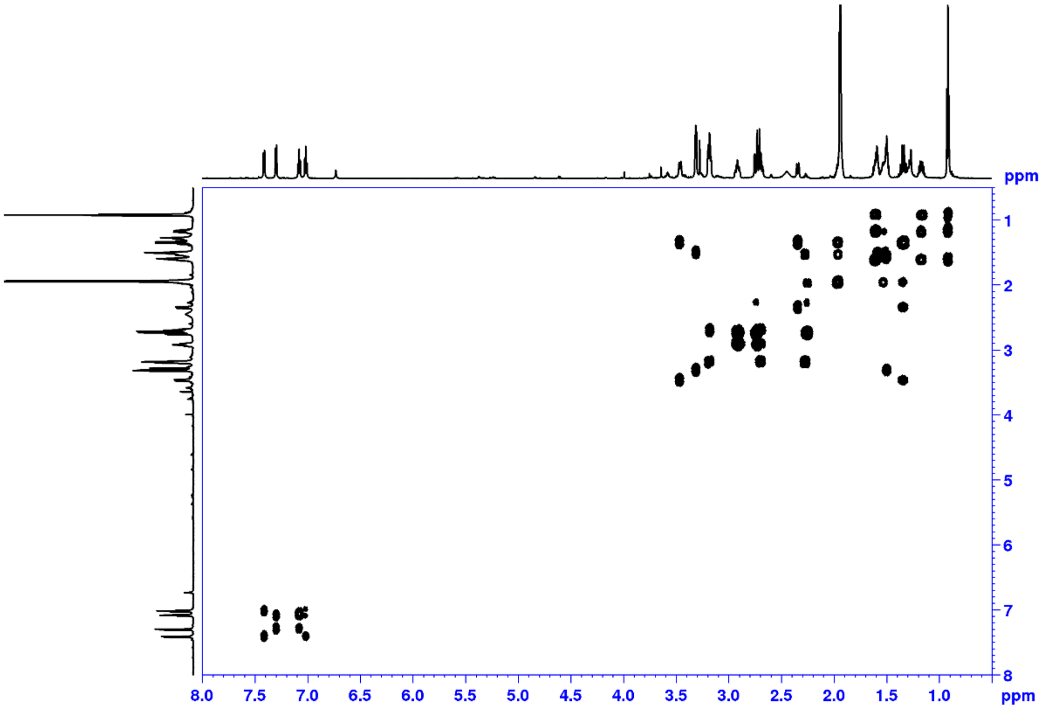

Figure S7: ^{1}H-^{1}H DQFCOSY of dihydrocorynantheal **2** with water suppression, magnitude mode processed, full range, in MeCN-d_3.

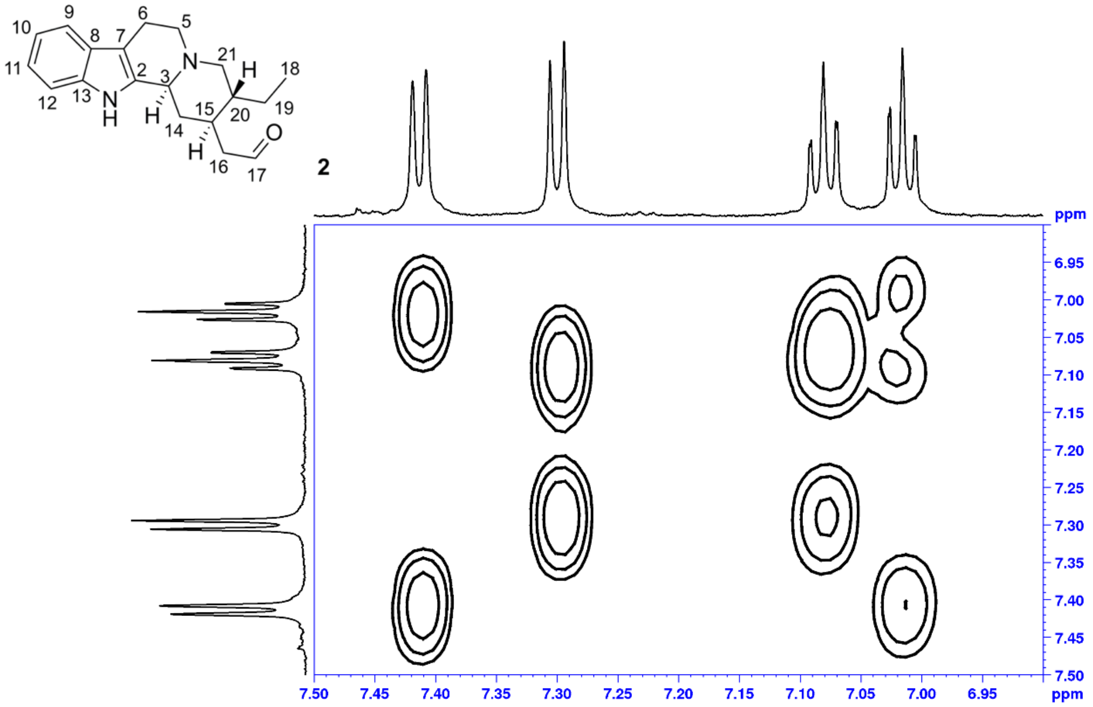

Figure S8: ^{1}H-^{1}H DQFCOSY of dihydrocorynantheal **2** with water suppression, magnitude mode processed, aromatic range, in MeCN-d_3.

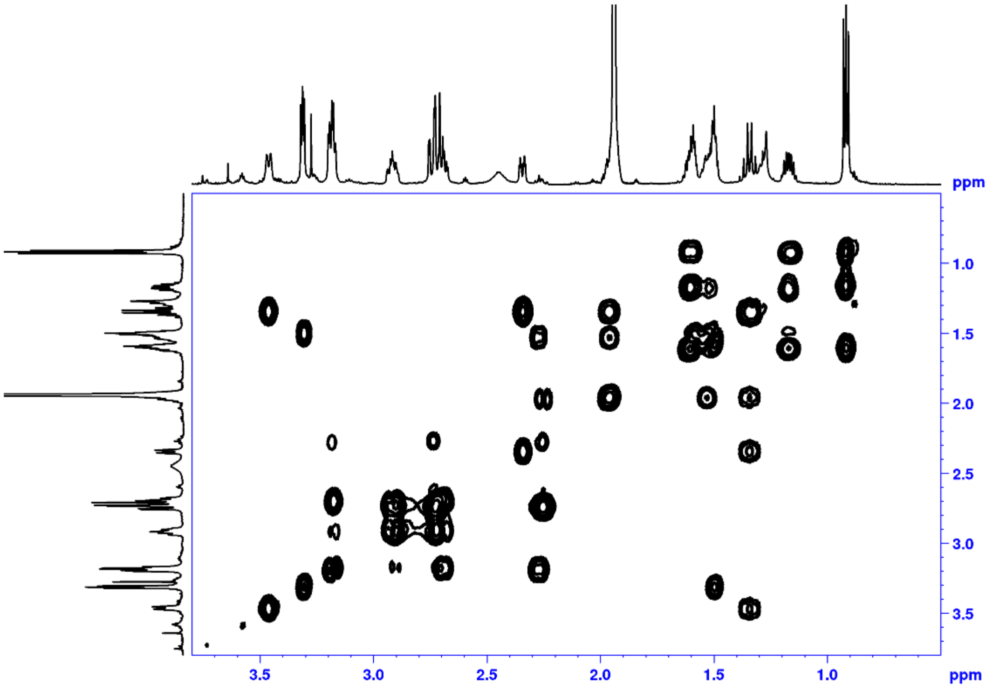

Figure S9: ^{1}H-^{1}H DQFCOSY of dihydrocorynantheal **2** with water suppression, magnitude mode processed, aliphatic range, in MeCN-d_3.

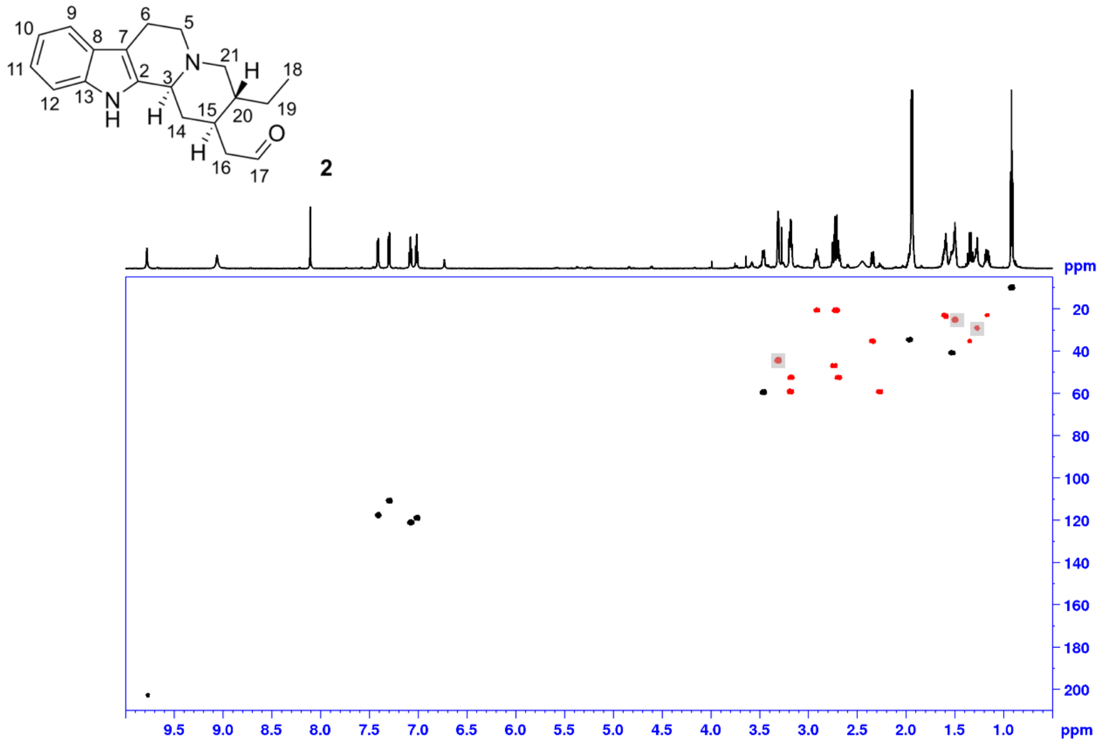

Figure S10: ^{1}H-^{13}C HSQC of dihydrocorynantheal **2**, full range. Shaded areas mark impurities, in MeCN-d_3.

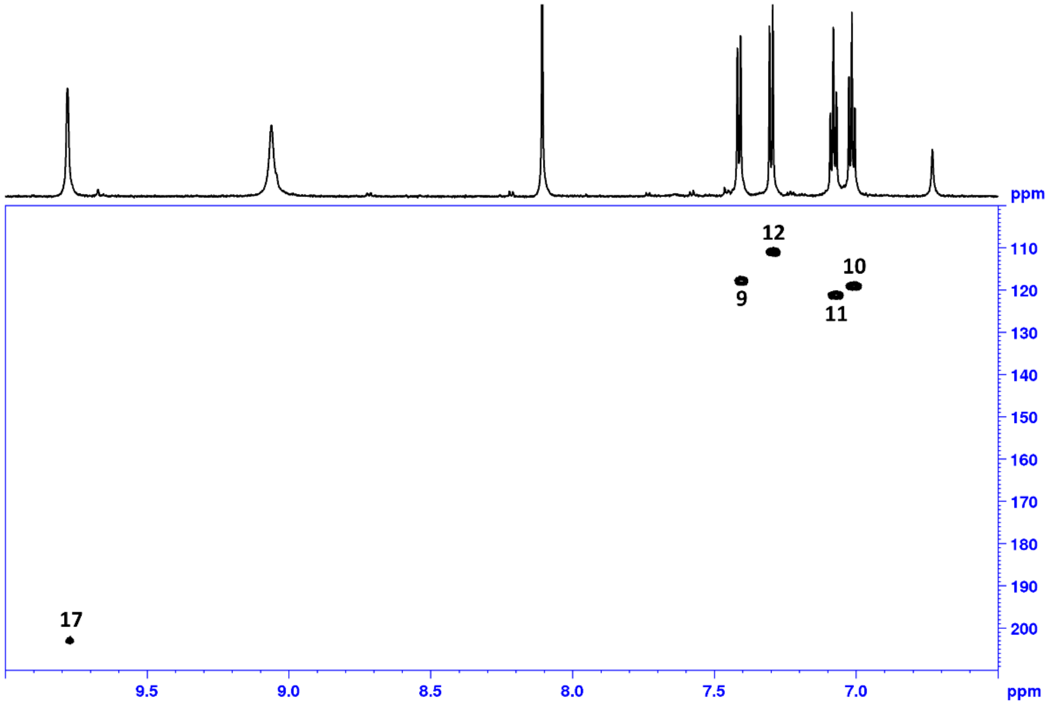

Figure S11: ^{1}H-^{13}C HSQC of dihydrocorynantheal **2**, aromatic range, in MeCN-d_3.

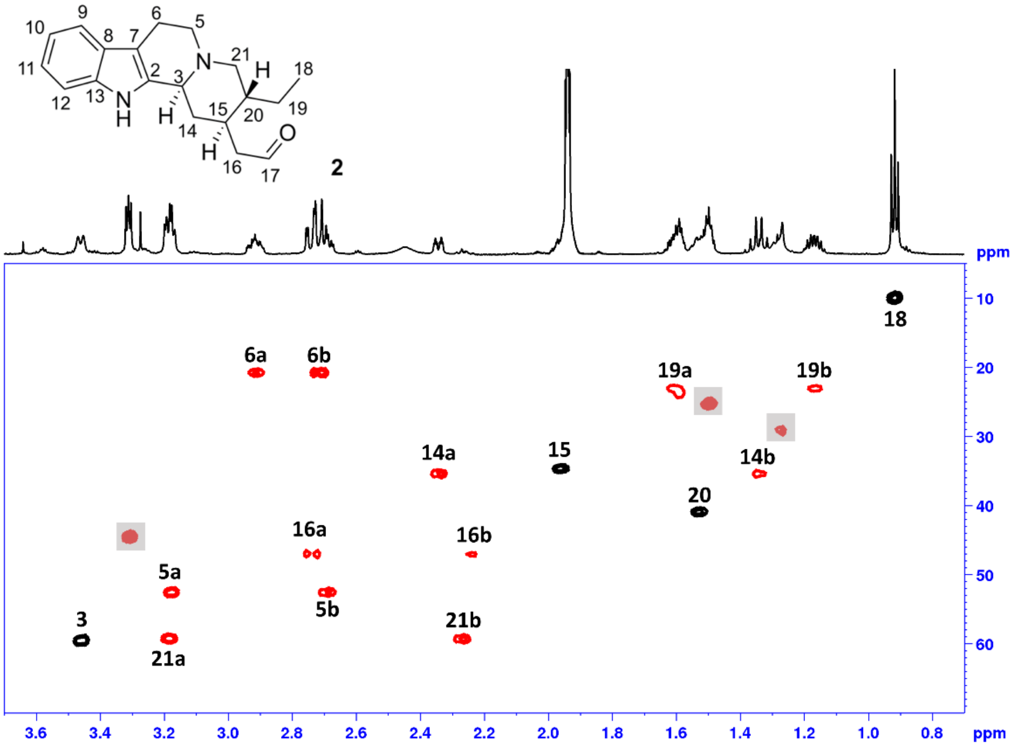

Figure S12: ^{1}H-^{13}C HSQC of dihydrocorynantheal **2**, aliphatic range. Shaded areas mark impurities, in MeCN-d_3.

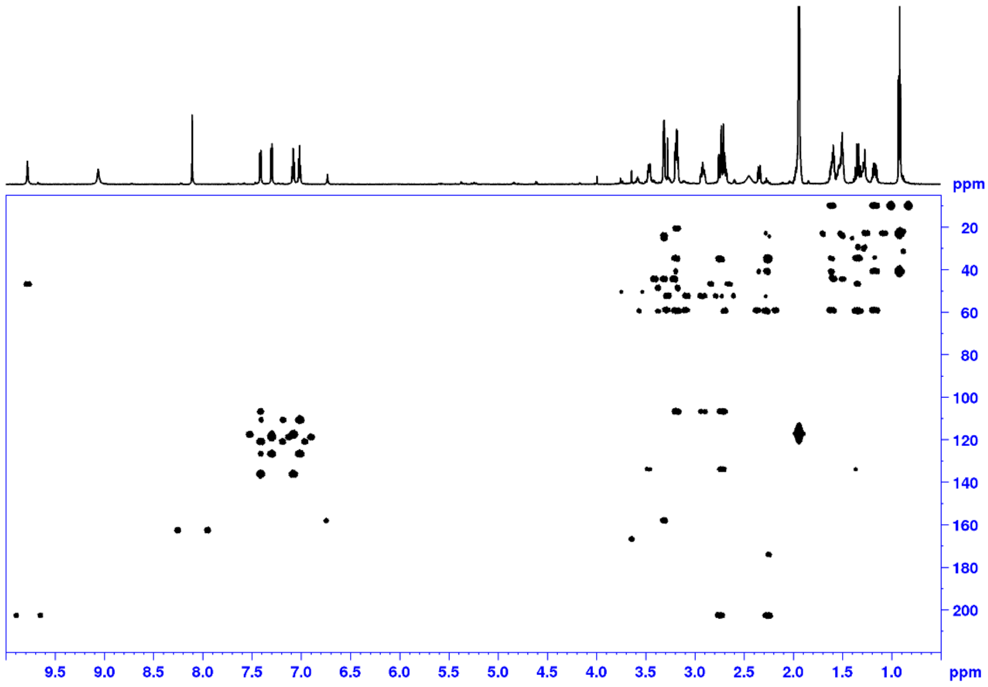

Figure S13: ^{1}H-^{13}C HMBC of dihydrocorynantheal **2**, full range, in MeCN-d_3

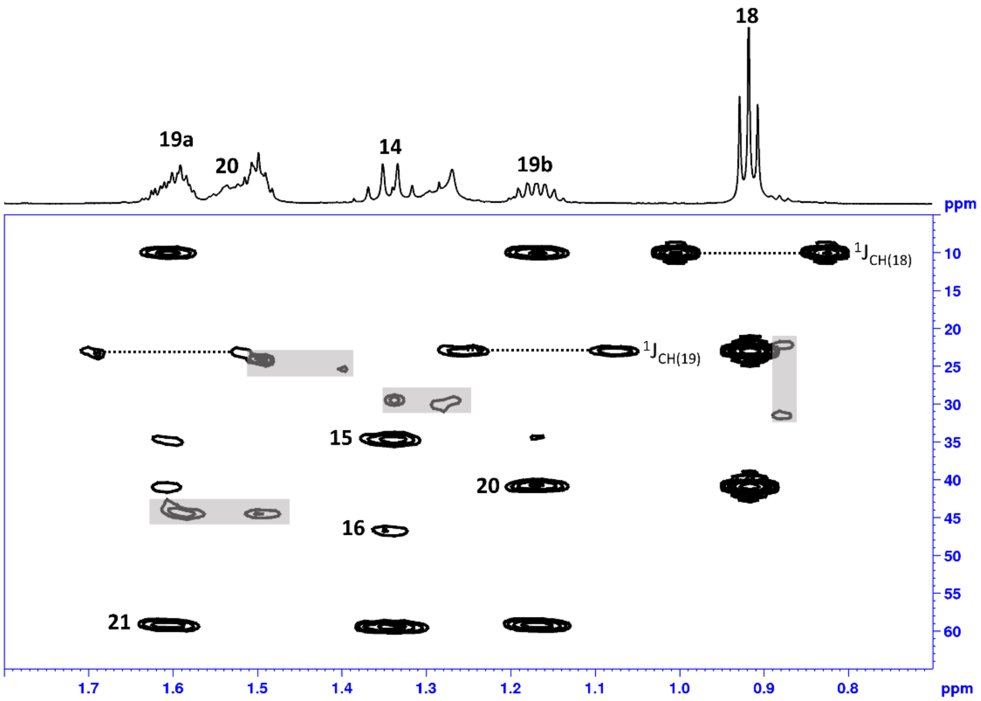

Figure S14: ^{1}H-^{13}C HMBC of dihydrocorynantheal **2**, detail, shaded area marks impurities, in MeCN-d_3.

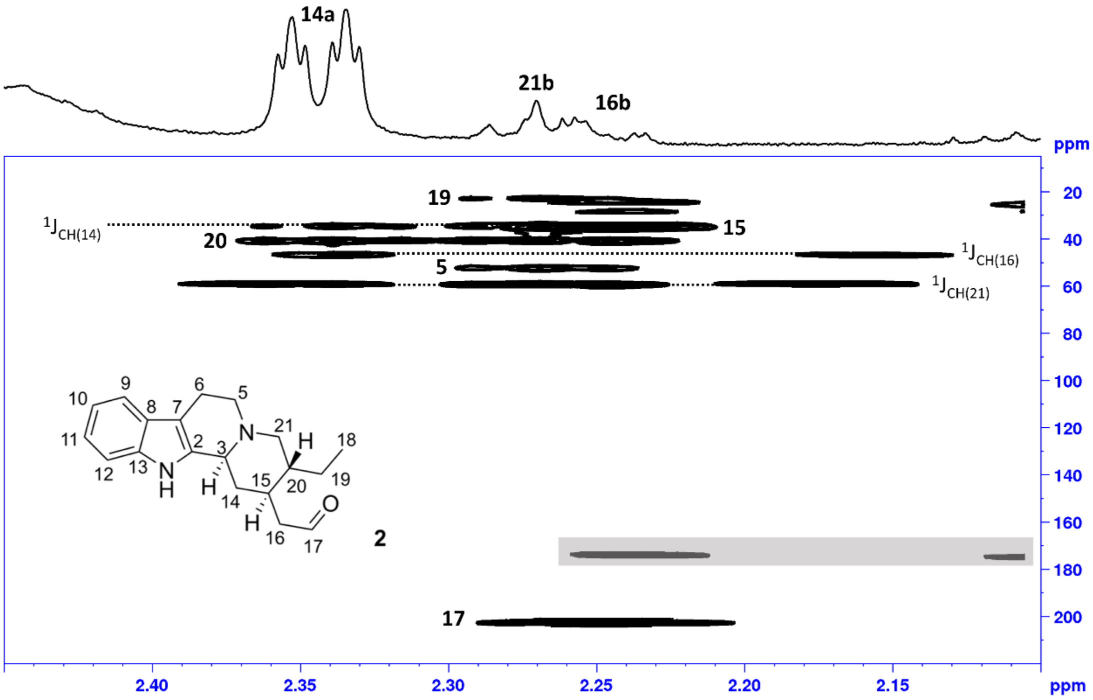

Figure S15: ^{1}H-^{13}C HMBC of dihydrocorynantheal **2**, detail, shaded area marks impurities, in MeCN-d_3.

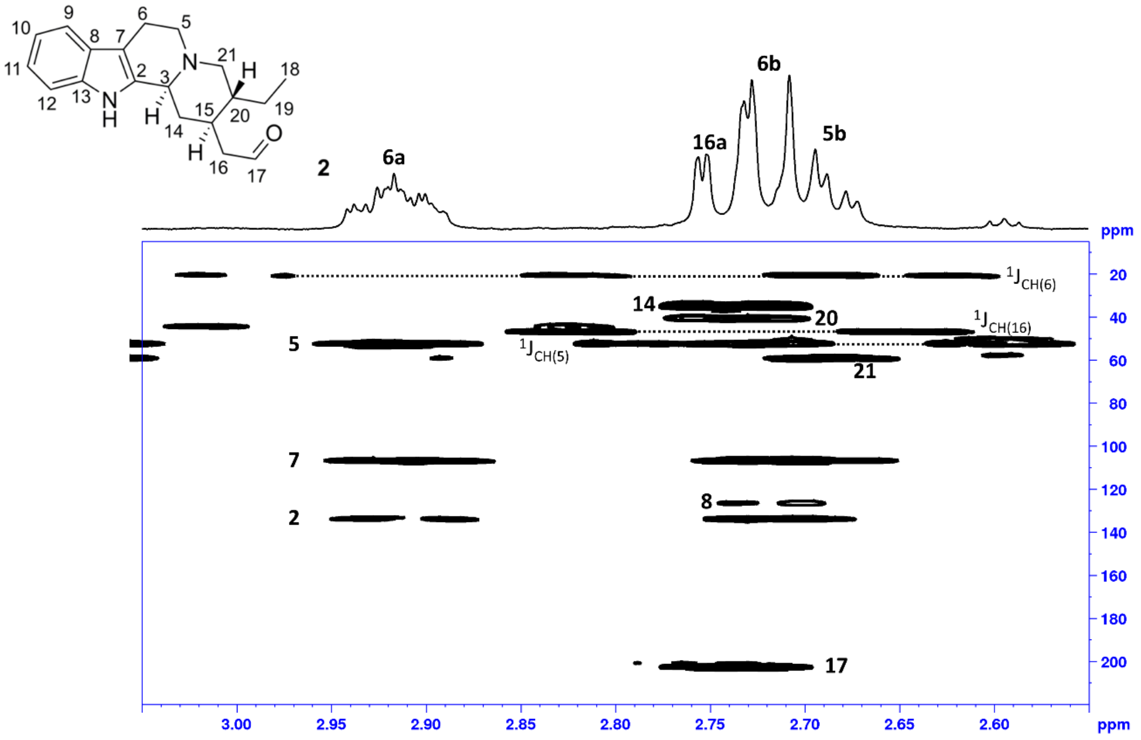

Figure S16: ^{1}H-^{13}C HMBC of dihydrocorynantheal **2**, detail, in MeCN-d_3.

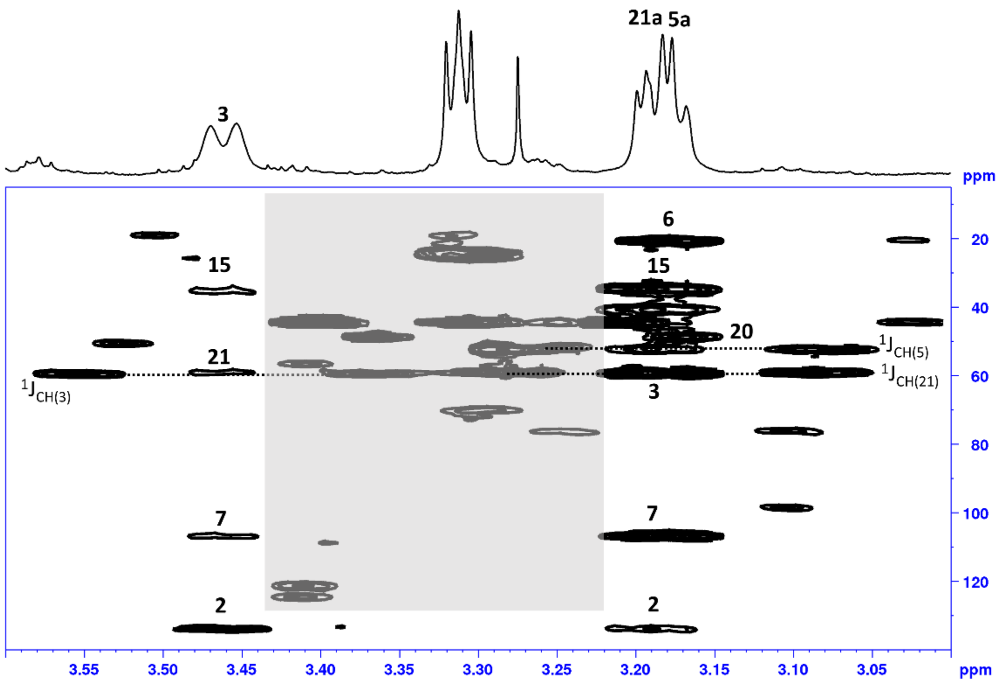

Figure S17: ^{1}H-^{13}C HMBC of dihydrocorynantheal **2**, detail, shaded area marks impurities, in MeCN-d_3.

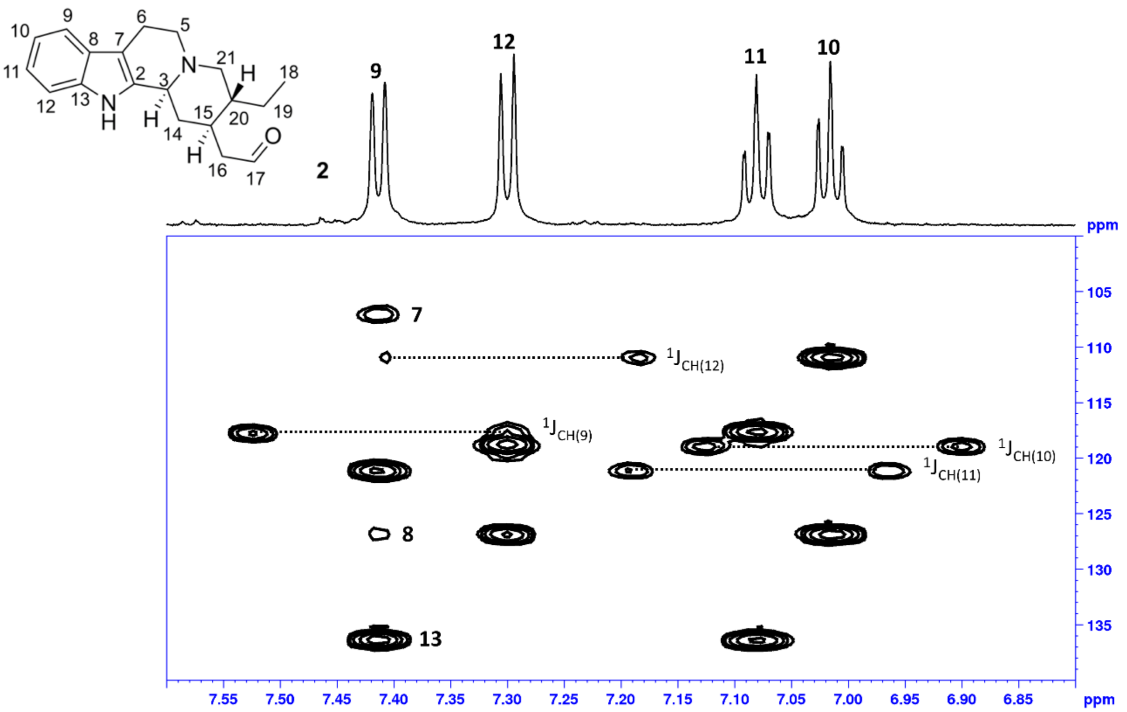

Figure S18: ^{1}H-^{13}C HMBC of dihydrocorynantheal **2**, detail, aromatic range, in MeCN-d_3.

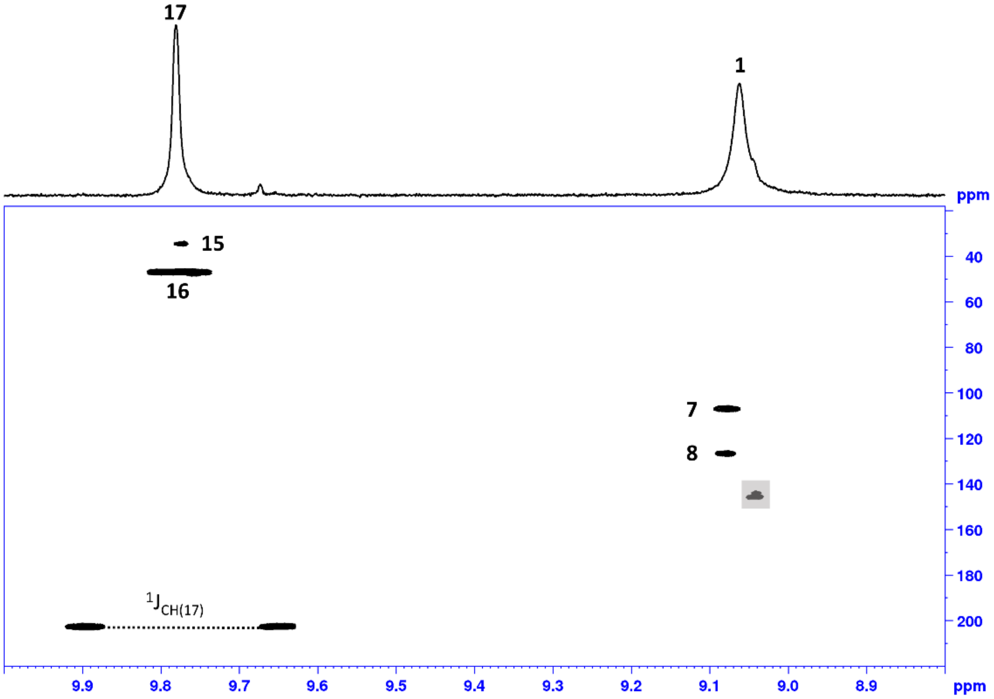

Figure S19: ^{1}H-^{13}C HMBC of dihydrocorynantheal **2**, detail, shaded area marks impurities, in MeCN-d_3.

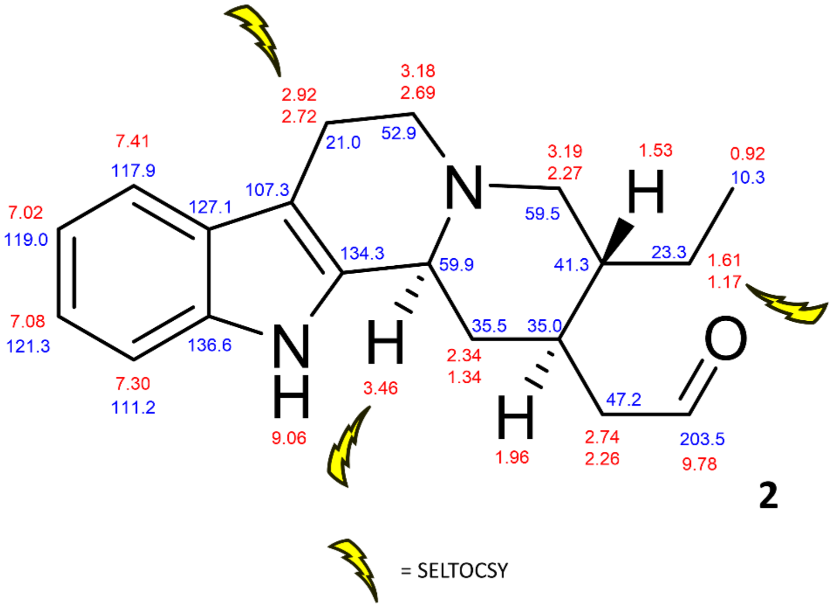

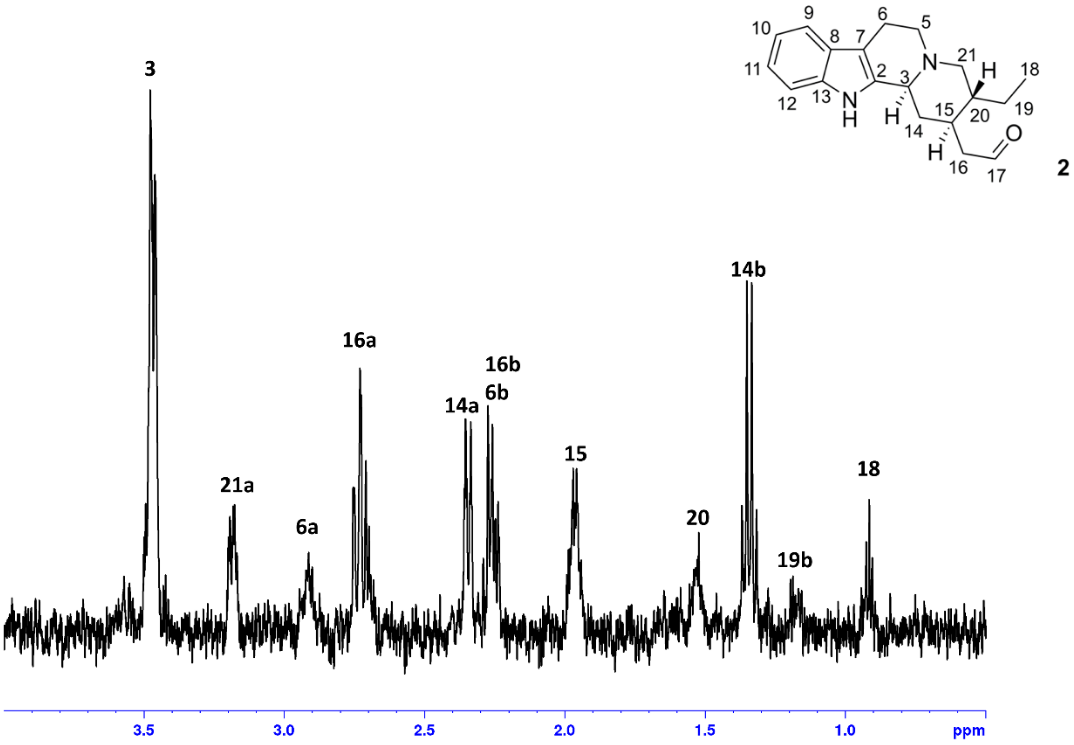

Figure S20: ^{1}H-^{1}H SELTOCSY of dihydrocorynantheal **2**, transmitter on H-3, 120 ms mixing time, in MeCN-d_3.

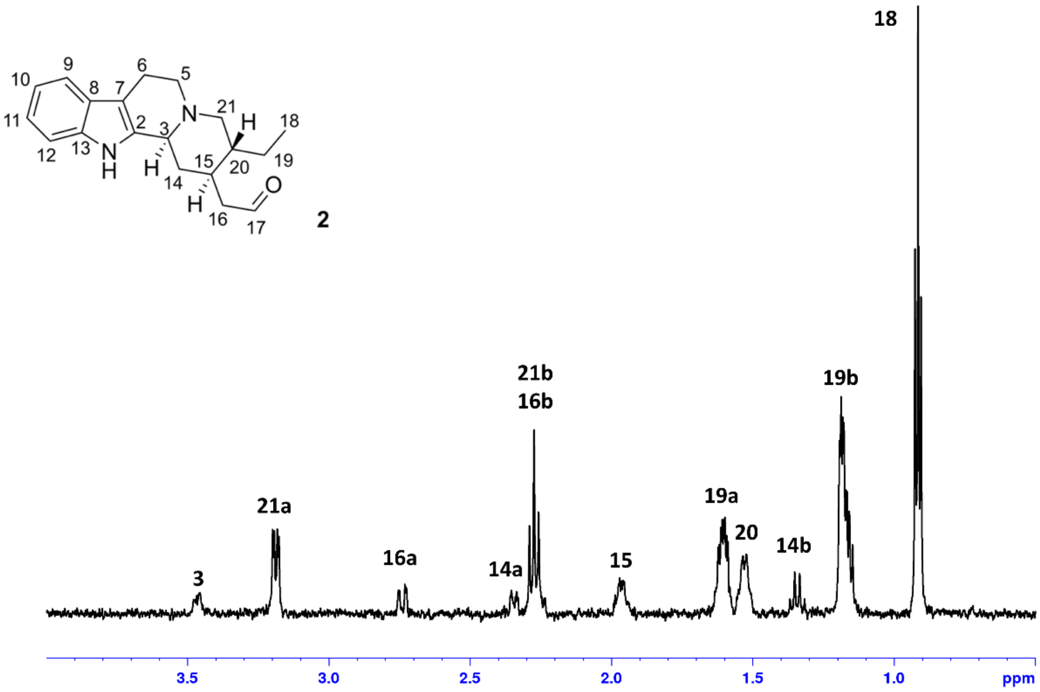

Figure S21: ^{1}H-^{1}H SELTOCSY of dihydrocorynantheal **2**, transmitter on H-19b, 120 ms mixing time, in MeCN-d_3.

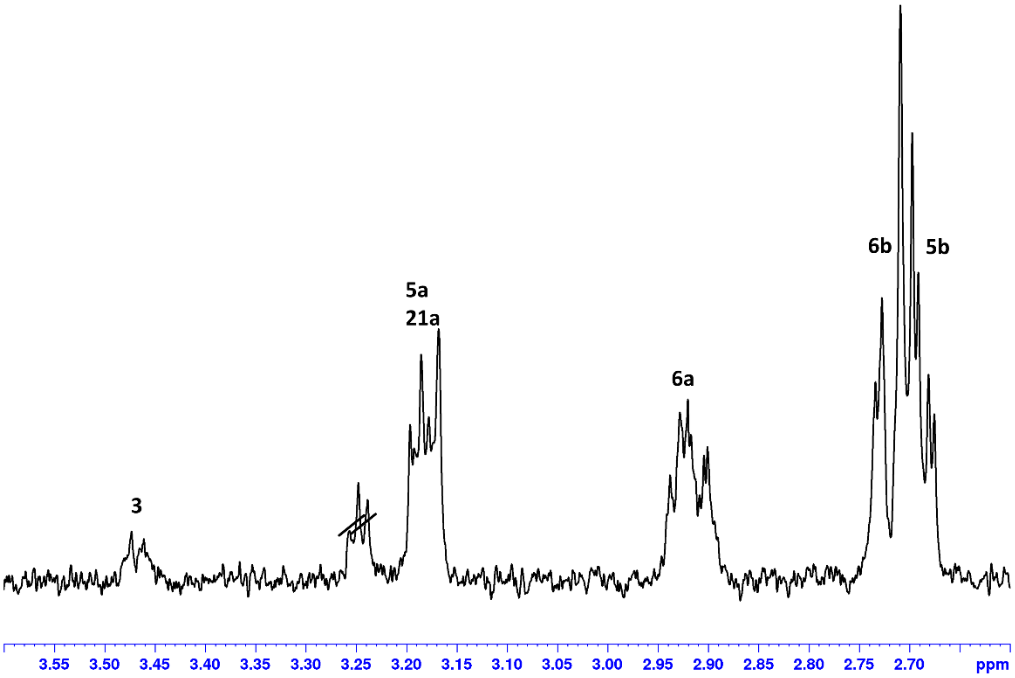

Figure S22: ^{1}H-^{1}H SELTOCSY of dihydrocorynantheal **2**, transmitter on H-6a, 120 ms mixing time, in MeCN-d_3.

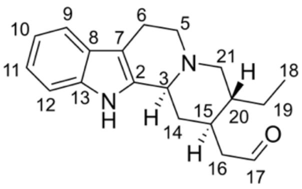

2

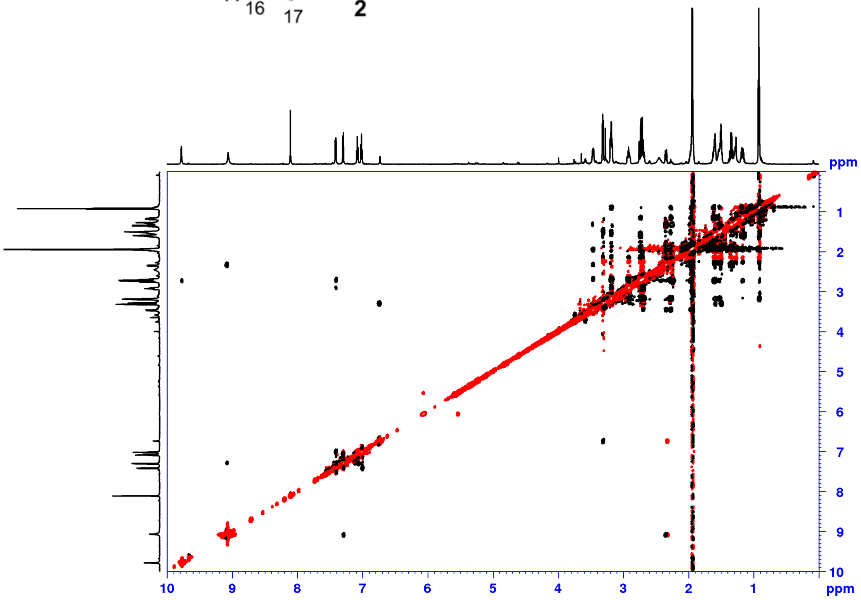

Figure S23: ^1H-^1H ROESY of dihydrocorynantheal **2**, with water suppression, spin lock 400 ms, in MeCN-d_3.

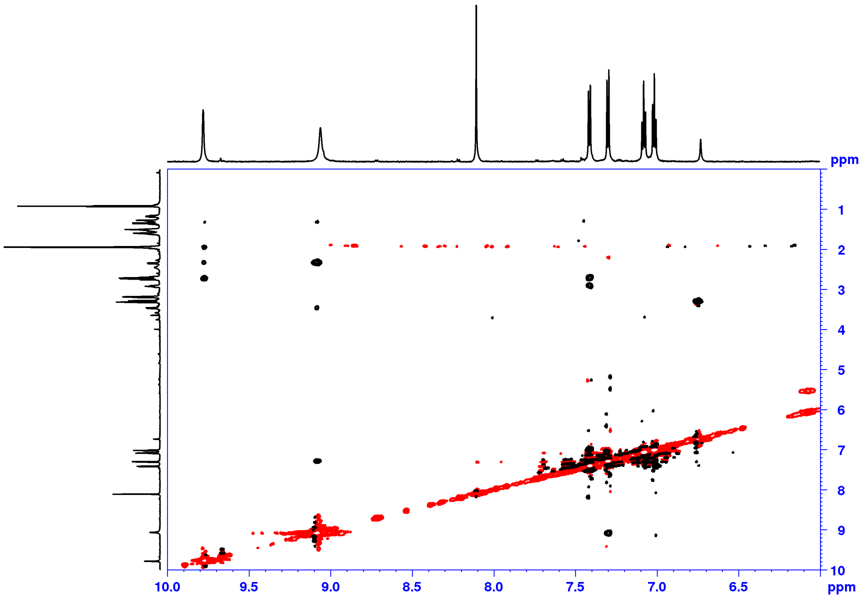

Figure S24: ^1H-^1H ROESY of dihydrocorynantheal **2**, with water suppression, detail, spin lock 400 ms, in MeCN-d_3.

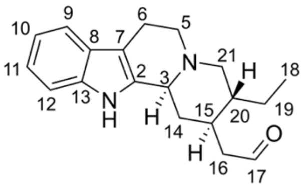

2

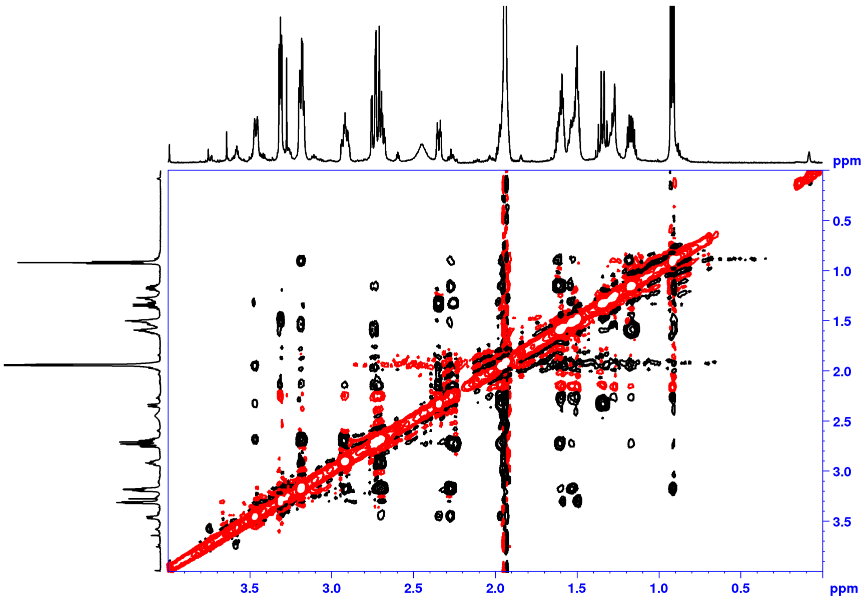

Figure S25: ^{1}H-^{1}H ROESY of dihydrocorynantheal **2**, with water suppression, detail, spin lock 400 ms, in MeCN-d_3.

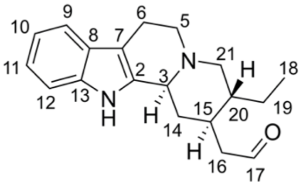

2

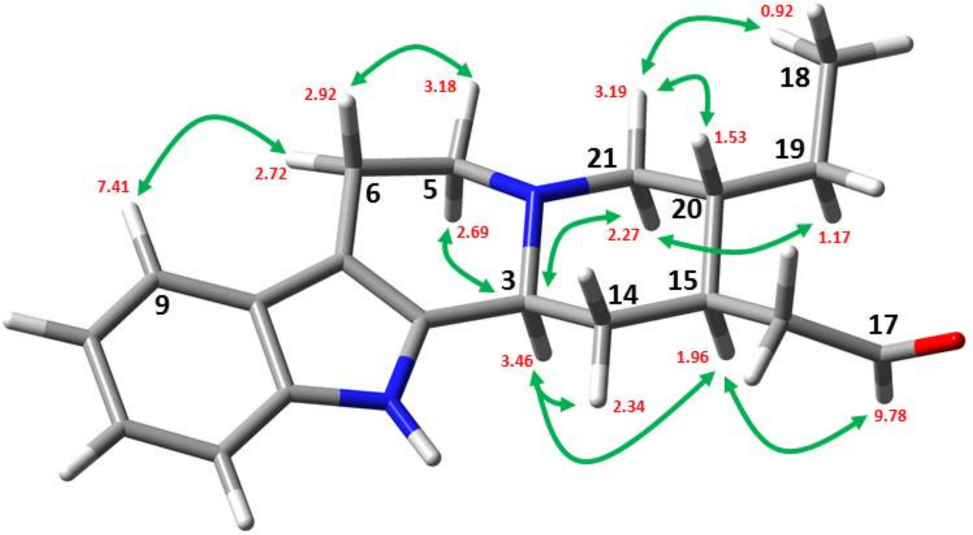

Figure S26: Structure of dihydrocorynantheal **2** optimized using Gaussian 16W (PM6, solvent MeCN). Important ROESY correlations are depicted in green. The assignment is based on definition of all alpha-oriented protons starting from H-3. The orientation of H-20 was deduced from the correlation H-21b→H-20. The configuration of position 6 was assigned from ROESY correlations to H-9 and H-5β.

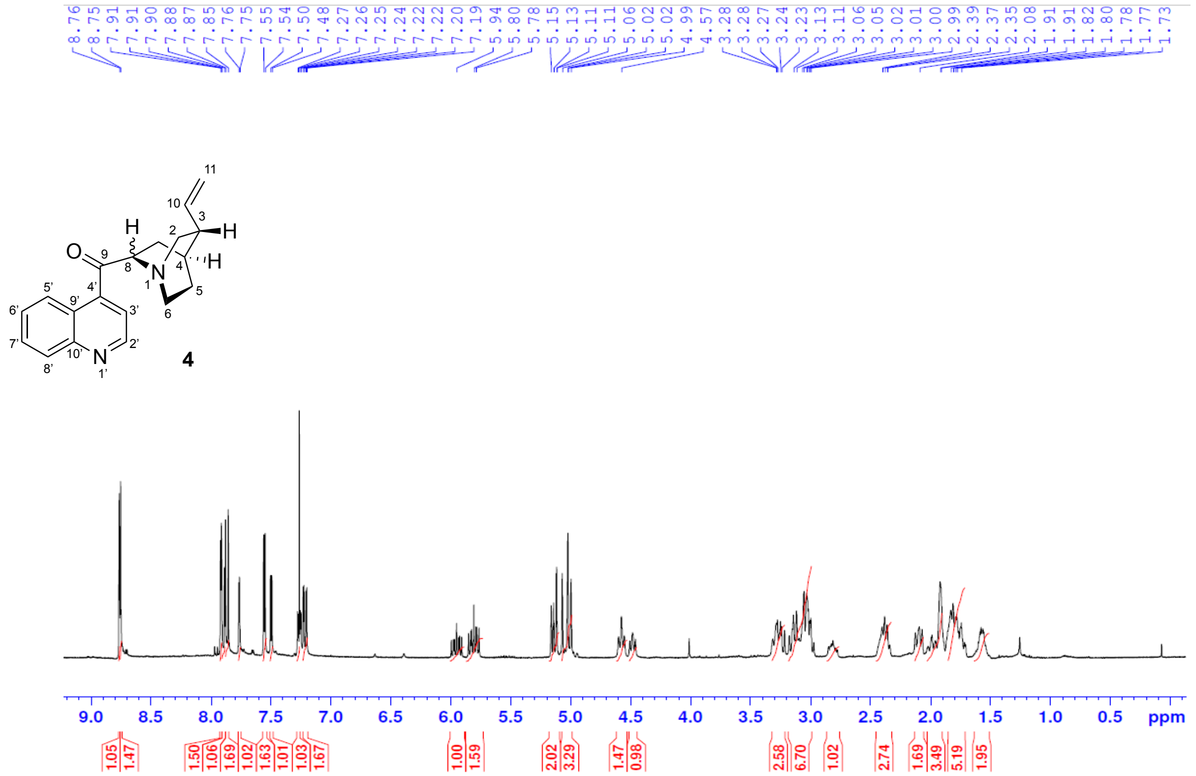

Figure S28: ^{1}H-NMR of 6´-hydroxycinchoninone **4**, 400 MHz, $CDCl_3$, 298 K

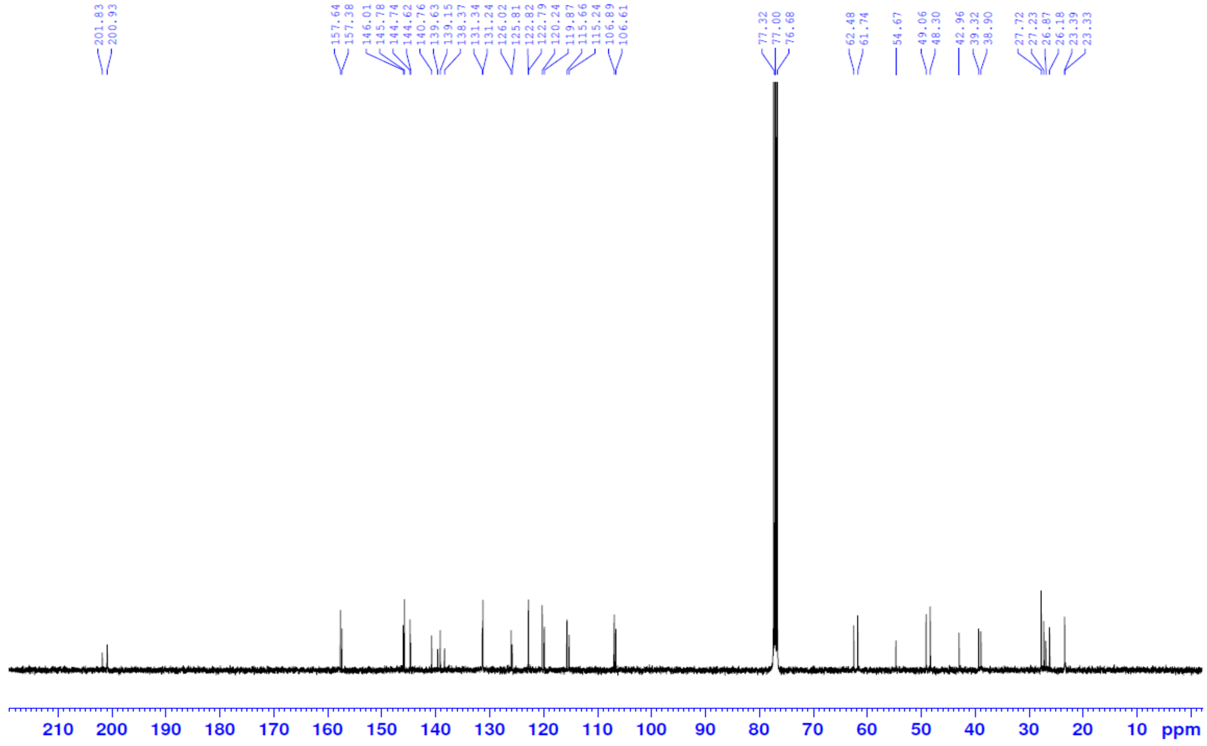

Figure S29: ^{13}C-NMR of 6´-hydroxycinchoninone **4**, 101 MHz, $CDCl_3$, 298 K

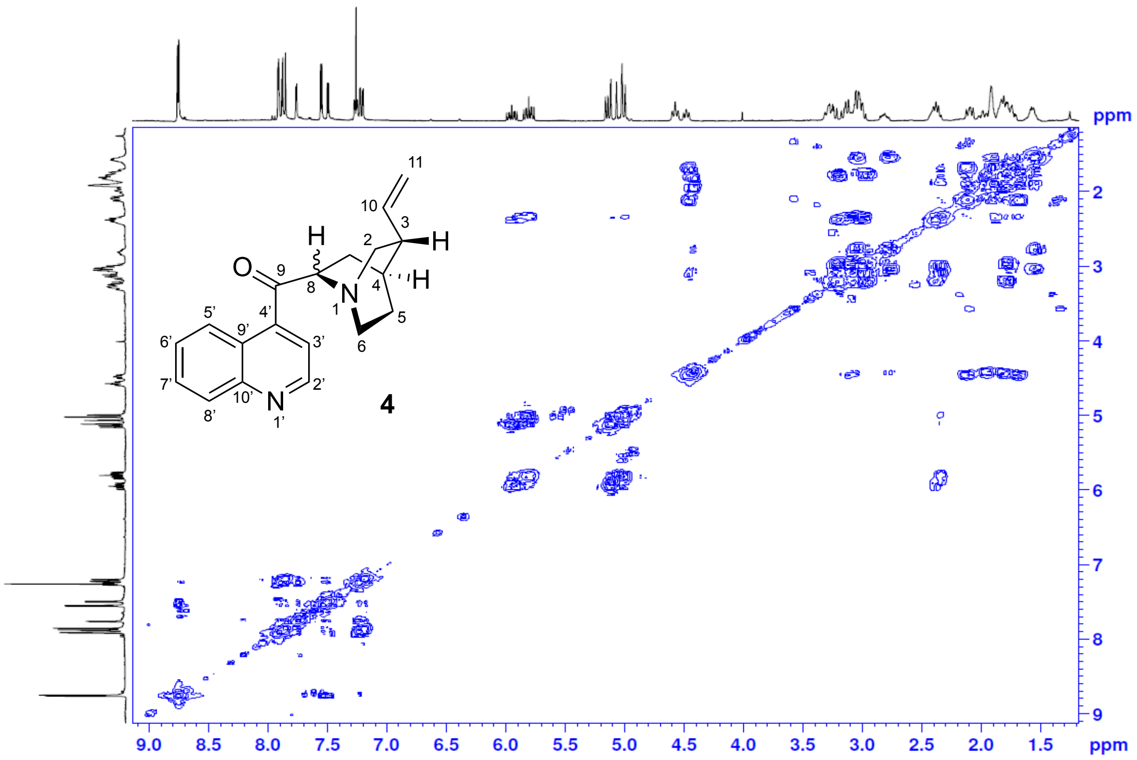

Figure S30: ^{1}H-^{1}H COSY of 6′-hydroxycinchoninone **4**, 400 MHz, $CDCl_3$, 298 K

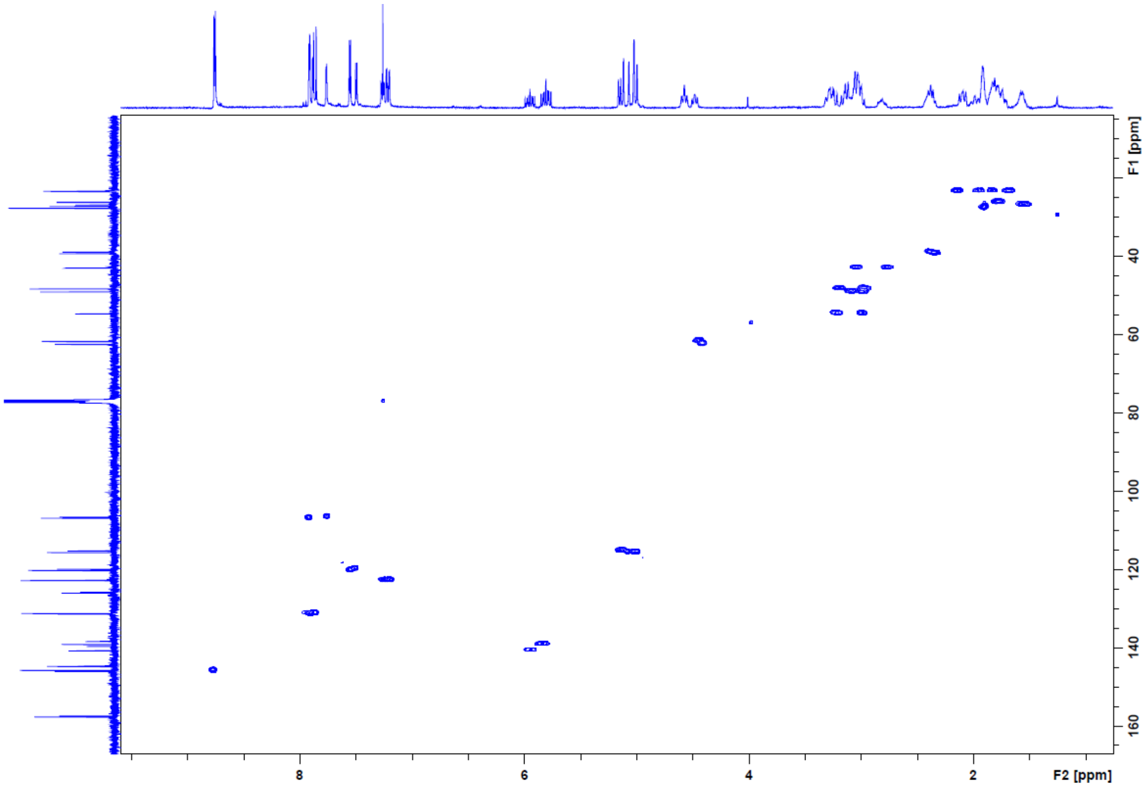

Figure S31: HSQC of 6′-hydroxycinchoninone **4**, 400 MHz, $CDCl_3$, 298 K

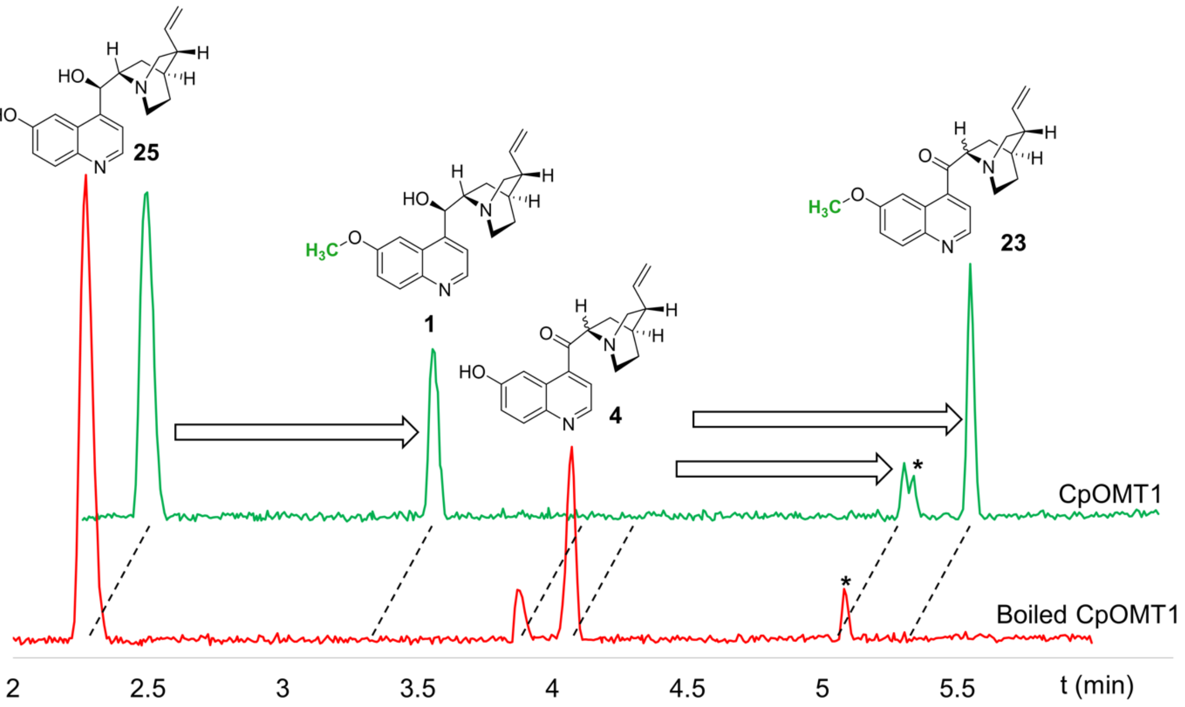

Figure S32: TIC traces of competition assay between substrates 25 and 4 incubated with CpOMT1 for 12 hours. Notice that all ketone analogs are in an inseparable tautomeric equilibrium, as indicated by the two peaks in the chromatogram. * indicates an unrelated impurity.

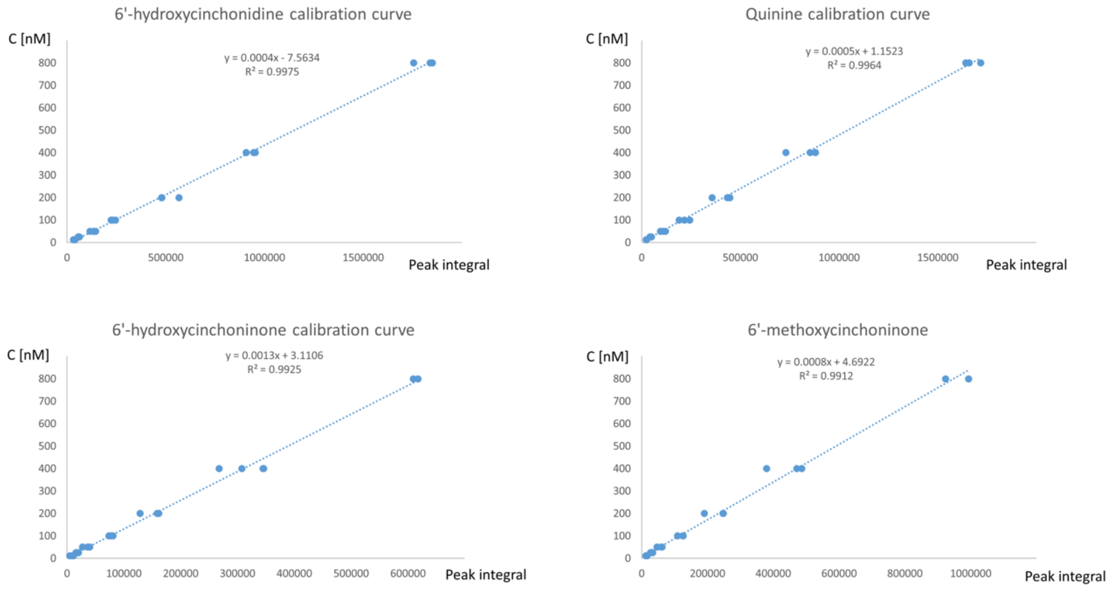

Figure S33: calibration curves for substrates and products of CpOMT1.

Table S1. Quantifier and qualifier *m/z* fragments used to calculate CpOMT1 product conversion

Compound	Precursor ion *m/z*	Quantifier *m/z* [collision energy]	Qualifier *m/z* [collision energy]
Quinine	163.1	188.9 [15.0 V]	159.9 [22.0 V]; 173.9 [23.0 V]; 210.9 [12.0 V]
6'-methoxycinchoninone	162.1	159.0 [19.0 V]	214.0 [13.0 V]; 67.2 [16.0 V]; 115.0 [13.0 V]
6'-hydroxycinchonidine	156.1	174.9 [16.0 V]	145.9 [26.0 V]; 202.9 [12.0 V]; 67.2 [15.0 V]
6'-hydroxycinchoninone	155.1	145.0 [22.0 V]	200.9 [12.0 V]; 67.2 [16.0 V]; 108.1 [13.0 V]

Table S2. Primer list

Primers	Primer sequence	Gene function
pOPINF seq fw	CGGGACCTTTAATTCAACCCAAC	Sequencing and colony PCR
pOPINF seq re	CACCACCTTCTGATAGGCAG	
pESC seq fw	GGTGGTAATGCCATGTAATATG	
pESC seq re	GGCAAGGTAGACAAGCCGACAAC	
pOPINM seq fw	GAAATCATGCCGAACATC	
pOPINM seq re	TAGCCAGAAGTCAGATGCT	
pOPINF-Cp2794.con4 fw	AAGTTCTGTTTCAGGGCCCGGCAAAAACACCAGCAGAAACA	MDHs
pOPINF-Cp2794.con4 re	ATGGTCTAGAAAGCTTTAAGCTGATTTCAATGAATTC	
pOPINF-Cp39479 fw	AAGTTCTGTTTCAGGGCCCGGCTTCATCACCAGAGTTGAA	
pOPINF-Cp39479 re	ATGGTCTAGAAAGCTTTAAGTAGATTTTAGCGTATTCCC	
pOPINF-Cp1238 fw	AAGTTCTGTTTCAGGGCCCGGCGACTACTCTAAACAGCG	
pOPINF-Cp1238 re	ATGGTCTAGAAAGCTTTACGCATACAAATCTATTCCC	
pOPINF-Cp13924 fw	AAGTTCTGTTTCAGGGCCCGGCAGAGTCTTACGAGGAAGAG	
pOPINF-Cp13924 re	ATGGTCTAGAAAGCTTTAGGCAGCCTTCAATGTGT	
pOPINF-Cp10715 fw	AAGTTCTGTTTCAGGGCCCGGCTGGAAAATCCCCAGA	
pOPINF-Cp10715 re	ATGGTCTAGAAAGCTTTAAGGAGCTTTCAAGGTGTTG	
pOPINF-Cp14456.con2 fw	AAGTTCTGTTTCAGGGCCCGGCAAAATCACCTGAAATGG	
pOPINF-Cp14456.con2 re	ATGGTCTAGAAAGCTTTAAGAATTACCATTCATATCAAAAGC	
pOPINF-Cp15915 fw	AAGTTCTGTTTCAGGGCCCGGCTGAAAAACCAGCATT	
pOPINF-Cp15915 re	ATGGTCTAGAAAGCTTTAATCACATTTAAGCGTGTTTC	
pOPINF-Cp640 fw	AAGTTCTGTTTCAGGGCCCGGCTGGGCAGAATTTAAGG	
pOPINF-Cp640 re	ATGGTCTAGAAAGCTTTAAGTGCTTGGGGTAGCC	
pOPINF-Cp1494 fw	AAGTTCTGTTTCAGGGCCCGATTTTTTTGTCTCTCAATACATC	
pOPINF-Cp1494 re	ATGGTCTAGAAAGCTTTAGAGTGCCTTCAAAGTATTTG	
pOPINF-Cp1348 fw	AAGTTCTGTTTCAGGGCCCGCCAGATAAAATGAAAATTTTTG	
pOPINF-Cp1348 re	ATGGTCTAGAAAGCTTTATATGTCACCATCCCAAAGTT	

pOPINF-Cp19597 fw	AAGTTCTGTTTCAGGGCCCGCCAGATAAAATGAAAATTTTTG	
pOPINF-Cp19597 re	ATGGTCTAGAAAGCTTTATTTAATTATGTGTATGCCATG	
pOPINF-Cp394 fw	AAGTTCTGTTTCAGGGCCCGGCTGTCGAGTCTCAAATTTT	
pOPINF-Cp394 re	ATGGTCTAGAAAGCTTTATTCATCCCAGATGTCCTC	
pOPINF-Cp12411 fw	AAGTTCTGTTTCAGGGCCCGGCTACAATTAGAGAGGTAACCT	
pOPINF-Cp12411 re	ATGGTCTAGAAAGCTTTAGACCTCTGCGTCCAAC	
pOPINF-Cp6072 fw	AAGTTCTGTTTCAGGGCCCGGCGGCGAAAGATATTCG	
pOPINF-Cp6072 re	ATGGTCTAGAAAGCTTTAGATATCACCATCCCAGAGA	
pOPINF-Cp5024 fw	AAGTTCTGTTTCAGGGCCCGGCCACTGGGATAAAGTACT	
pOPINF-Cp5024 re	ATGGTCTAGAAAGCTTTAGACCTCACCATCCCAG	
pOPINF-CpDCS fw	AAGTTCTGTTTCAGGGCCCGGCCGGAAAATCTCAAGAA	
pOPINF-CpDCS re	ATGGTCTAGAAAGCTTTAAGCAGATTTTAAGGTATTGCC	
pESC-Cp2088.con3 fw	ACCCTCACTAAAGGGCGGCCGCAACCATGAAAATGGAATTTTCCTTC	Cytochromes P450
pESC-Cp2088.con3 re	GTCATCCTTGTAATCCATCGATACAAGGGAAGGATAATACAATGTG	
pESC-Cp10558.con5 fw	ACCCTCACTAAAGGGCGGCCGCAACCATGCTTCTCTTTCCAGTTGAG	
pESC-Cp10558.con5 re	GTCATCCTTGTAATCCATCGATACATTAGGTAGACTTGCAACAAG	
pESC-Cp1125.con6 fw	ACCCTCACTAAAGGGCGGCCGCAACCATGAAGGAAAAACAAGAAAGG	
pESC-Cp1125.con6 re	GTCATCCTTGTAATCCATCGATACCAACTTCACTGGAATAGCC	
pESC-Cp4378 fw	ACCCTCACTAAAGGGCGGCCGCAACCATGGCAAATTCCTCAAGG	
pESC-Cp4378 re	GTCATCCTTGTAATCCATCGATACAGAAAGTGGAGTTGGGATAG	
pESC-Cp27708 fw	ACCCTCACTAAAGGGCGGCCGCAACCATGGCTGTAGAAATTTCTCTC	
pESC-Cp27708 re	GTCATCCTTGTAATCCATCGATACATAAAGATGGGGAAACAGAC	
pESC-Cp8105.con1 fw	ACCCTCACTAAAGGGCGGCCGCAACCATGGGGCTCATGGTTTC	
pESC-Cp8105.con1 re	GTCATCCTTGTAATCCATCGATACTTTAAAAAAAGAATGACGATACA	
pESC-Cp12022.con2 fw	ACCCTCACTAAAGGGCGGCCGCAACCATGGAGTTCCTCACAACC	
pESC-Cp12022.con2 re	GTCATCCTTGTAATCCATCGATACTTTCAAATAGGAATGGCTATG	
pESC-Cp24354 fw	ACCCTCACTAAAGGGCGGCCGCAACCATGAGAACATATATTTCTCTAATC	
pESC-Cp24354 re	GTCATCCTTGTAATCCATCGATACCAAAGCAGAAGGACGATT	
pESC-Cp28986 fw	ACCCTCACTAAAGGGCGGCCGCAACCATGGAATTTATTCAGTTCTCAG	
pESC-Cp28986 re	GTCATCCTTGTAATCCATCGATACATTAATGGGAAGAGGAATCAC	
pESC-Cp620.con9 fw	ACCCTCACTAAAGGGCGGCCGCAACCATGGAGGAGCTTGATCAAA	
pESC-Cp620.con9 re	GTCATCCTTGTAATCCATCGATACGTCATCCAGGAAATCATGATA	
pESC-Cp7234 fw	ACCCTCACTAAAGGGCGGCCGCAACCATGGAGCTCCAAAATTTC	
pESC-Cp7234 re	GTCATCCTTGTAATCCATCGATACTATAGCAACGGCAGGATC	
pESC-Cp16649 fw	ACCCTCACTAAAGGGCGGCCGCAACCATGCTATCAAGTTCTTCAAAC	
pESC-Cp16649 re	GTCATCCTTGTAATCCATCGATACAAATCGACTCATTGGAGTTG	
pESC-Cp7253.con4 fw	ACCCTCACTAAAGGGCGGCCGCAACCATGGAGAATTTTACAGTTGAAG	
pESC-Cp7253.con4 re	GTCATCCTTGTAATCCATCGATACATTCTTGATCATTGTAGGAAC	
pESC-Cp8609 fw	ACCCTCACTAAAGGGCGGCCGCAACCATGGCCATGGAAGTCC	
pESC-Cp8609 re	GTCATCCTTGTAATCCATCGATACAAGTTGAGAAATGAGGGAACT	
pESC-Cp3825.con6 fw	ACCCTCACTAAAGGGCGGCCGCAACCATGATGAATTCCCTCATTG	

pESC-Cp3825.con6 re	GTCATCCTTGTAATCCATCGATACGTAGAGTGGACAAAGCAAAC	
pESC-Cp12767 fw	ACCCTCACTAAAGGGCGGCCGCAACCATGATGTTTCAGATCTTAATTAACA	
pESC-Cp12767 re	GTCATCCTTGTAATCCATCGATACCTCATAGAGTGATGCATCAG	
pESC-Cp35516 fw	ACCCTCACTAAAGGGCGGCCGCAACCATGGATGCCCTGACG	
pESC-Cp35516 re	GTCATCCTTGTAATCCATCGATACCAGAGGGAGTGGGACAG	
pESC-Cp1963.con4 fw	ACCCTCACTAAAGGGCGGCCGCAACCATGGCAACAAATTCATTAAC	
pESC-Cp1963.con4 re	GTCATCCTTGTAATCCATCGATACTTTCCTCTTTTTCAACGTAAC	
pESC-Cp17862 fw	ACCCTCACTAAAGGGCGGCCGCAACCATGCTTGGCCATGCC	
pESC-Cp17862 re	GTCATCCTTGTAATCCATCGATACCTCATAACATGCTGGACCA	
pESC-Cp2392.con6 fw	ACCCTCACTAAAGGGCGGCCGCAACCATGATGATGTTGAGCATTTTC	
pESC-Cp2392.con6 re	GTCATCCTTGTAATCCATCGATACTCCATGTAATCGGTAAGTAGG	
pOPINF-Cp23298 fw	AAGTTCTGTTTCAGGGCCCGGATCCAGCAATTCCAA	Esterases
pOPINF-Cp23298 re	ATGGTCTAGAAAGCTTTAGATATATTTATCAGTTATTTCTTG	
pOPINF-Cp10039 fw	AAGTTCTGTTTCAGGGCCCGGAAGGTGAGAAGCAGCA	
pOPINF-Cp10039 re	ATGGTCTAGAAAGCTTTAATTAAATGGAACCAAAGTC	
pOPINF-Cp23301 fw	AAGTTCTGTTTCAGGGCCCGTTTTGCGTTACTTACACTTCA	
pOPINF-Cp23301 re	ATGGTCTAGAAAGCTTTAGATGTGTTCATCAACTATCTCT	
pOPINF-Cp40444 fw	AAGTTCTGTTTCAGGGCCCGGAAATGGTGAAGCAGAC	
pOPINF-Cp40444 re	ATGGTCTAGAAAGCTTTAATTAGGAGGGTTTTGATCC	
pOPINF-Cp7988 fw	AAGTTCTGTTTCAGGGCCCGATATTTCAAGTCAATTGGTCA	
pOPINF-Cp7988 re	ATGGTCTAGAAAGCTTTAATTACATCTCTCAGCAATTTC	
pOPINF-CpDCE fw	AAGTTCTGTTTCAGGGCCCGGAGAAACATTTTGTGCTAATC	
pOPINF-CpDCE re	ATGGTCTAGAAAGCTTTAGTCATATTTCTGGGCAATTT	
pOPINF-Cp10350.con2 fw	AAGTTCTGTTTCAGGGCCCGGATTTGGCCGGAAATG	OMTs
pOPINF-Cp10350.con2 re	ATGGTCTAGAAAGCTTTAATAATAATAAACCTCAATGAGAGA	
pOPINF-Cp3175.con4 fw	AAGTTCTGTTTCAGGGCCCGGAAAAAGACAAAATAGGAGGAG	
pOPINF-Cp3175.con4 re	ATGGTCTAGAAAGCTTTAAGGATAGGCCTCAATTACAG	
pOPINF-Cp11359 fw	AAGTTCTGTTTCAGGGCCCGCTAGCAACTTCTATACTACCAAAA	
pOPINF-Cp11359 re	ATGGTCTAGAAAGCTTTAGTTCTTGTACAATTCAATAACC	
pOPINF-Cp12638 fw	AAGTTCTGTTTCAGGGCCCGGACAAACAAGGAGAAGATGA	
pOPINF-Cp12638 re	ATGGTCTAGAAAGCTTTACTTGTAGAATTCCATCACAG	
pOPINF-Cp6036.con1 fw	AAGTTCTGTTTCAGGGCCCGGATTCTTTTGCAAAAGCAA	
pOPINF-Cp6036.con1 re	ATGGTCTAGAAAGCTTTACTTGTGGAACTCCATGATC	
pOPINF-Cp11992.con4 fw	AAGTTCTGTTTCAGGGCCCGGCAGAGGAGGAAGCTTG	
pOPINF-Cp11992.con4 re	ATGGTCTAGAAAGCTTTATTTGCATAGTTCCATGATCC	
pOPINF-CpOMT1 fw	AAGTTCTGTTTCAGGGCCCGGCAACATCTGAGAATTCTACTAA	
pOPINF-CpOMT1 re	ATGGTCTAGAAAGCTTTAAGGATAGACCTCAATGAGAC	

Table S3. Tabulated NMR values of dihydrocorynantheal 2

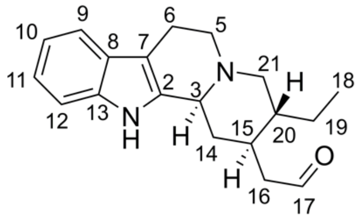

2

pos.	δ_H	*mult.*	J_{HH}	δ_C
1	9.06	s	-	-
2	-	-	-	134.3
3	3.46	*bd*	12.0	59.9
4	-	-	-	-
5a	3.18	*dd*	5.6/10.0	52.9
5b	2.69	*dd*	4.0/10.0	52.9
6a	2.92	*m*	-	21.0
6b	2.72	*d*	13.5	21.0
7	-	-	-	107.3
8	-	-	-	127.1
9	7.41	*d*	8.0	117.9
10	7.02	*ddd*	8.0/7.0/1.0	119.0
11	7.08	*ddd*	8.0/7.0/1.0	121.3
12	7.30	*d*	8.0	111.2
13	-	-	-	136.6
14a	2.34	*ddd*	3.0/3.2/12.0	35.5
14b	1.34	*q*	12.0	35.5
15	1.96	*m*	-	35.0
16a	2.74	*dd*	3.1/16.0	47.0
16b	2.26	*m*	-	47.0
17	9.78	*bs*	-	203.5
18	0.92	*t*	7.5	10.3
19a	1.60	*m*	-	23.3
19b	1.17	*m*	-	23.3
20	1.53	*m*	-	41.3
21a	3.19	*dd*	4.1/11.4	59.5
21b	2.27	*dd*	11.4/11.4	59.5

References

(1) Carmo, H. M.; Braz-Filho, R.; Vieira, I. J. C.; A novel alkaloid from aspidosperma pyricollum (Apocynaceae) and complete ^{1}H and ^{13}C chemical shift assignments. *Helv Chim Acta* **2015**, *98*, 1381-1386.

(2) Lichman, B. R.; Godden, G. T.; Hamilton, J. P.; Palmer, L.; Kamileen, M. O.; Zhao, D.; Vaillancourt, B.; Wood, J. C.; Sun, M.; Kinser, T. J.; Henry, L. K.; Rodriguez-Lopez, C.; Dudareva, N.; Soltis, D. E.; Soltis, P. S.; Buell, C. R.; O'Connor, S. E. The evolutionary origins of the cat attractant nepetalactone in catnip. *Sci Adv*. **2020**, *6*, eaba0721.

(3) Maisel, L; Fonseca, B.; Gonzales, S.; Baeza-Yates, R.; Cambiazo, V.; Campos, R.; Gonzales, M.; Orellana, A.; Retamales, J.; Silva, H. A rapid and efficient method for purifying high quality total RNA from peaches *Prunus persica* for functional genomics analyses. *Biol. Res.* **2005**, *38, 1*, 83-88.

(4) Berrow, N. S.; Alderton, D.; Sainsbury, S.; Nettleship, J.; Assenberg, R.; Rahman, N.; Stuart, D. I.; Owens, R. J. A versatile ligation-independent cloning method suitable for high throughput expression screening applications. *Nucleic Acids Res.* **2007**, *35,* e45.

(5) Gietz, R. D.; Schiestl, R. H. High-efficiency yeast transformation using the LiAc/SS carrier DNA/PEG method. *Nature Protocols* **2007**, *2,* 31-34.

(6) Leroux, S.; Larquetoux, L.; Nicolas, M.; Doris, E. Asymmetric Synthesis of (+)-Mequitazine from Quinine. *Org. Lett.* **2011**, *13*, 13, 3549–3551.

(7) Boratyn´ski, P. J.; Turowska-Tyrk, I.; Skarzewski, J. Synthetic approaches to 9-arylated Cinchona alkaloids: stereoselective addition of Grignard reagents to cinchonanones and hydroxylation of 9-phenylcinchonanes. *Tetrahedron: Asymmetry* **2012**, *23*, 876–883.

(8) Li, H.; Wang, Y.; Tang, L.; Deng, L. Highly Enantioselective Conjugate Addition of Malonate and β-Ketoester to Nitroalkenes: Asymmetric C−C Bond Formation with New Bifunctional Organic Catalysts Based on Cinchona Alkaloids. *J. Am. Chem. Soc.* **2004**, *126*, 32, 9906–9907.

(9) Smith, A. C.; Williams, R. M. Rabe Rest in Peace: Confirmation of the Rabe–Kindler Conversion of d-Quinotoxine Into Quinine: Experimental Affirmation of the Woodward–Doering Formal Total Synthesis of Quinine. *Angew. Chem. Intl. Ed.* **2008**, *47,* 1736 –1740.

IMAGE # 52623

ROYAL BOTANIC GARDENS KEW

NON COMMERCIAL
MATERIAL SUPPLY AGREEMENT FOR LIVE MATERIAL
(with effect from 1 January 2005)

The Royal Botanic Gardens, Kew ("Kew") is committed to the letter and spirit of the Convention on Biological Diversity ("CBD") and expects its partners to act in a manner consistent with the CBD. This agreement is designed to promote scientific research and exchange, whilst recognising the terms on which Kew acquired the plant or fungal material and the important role played by *ex situ* collections in the implementation of the CBD. Kew reserves the right not to supply any plant or fungal material if such supply would be contrary to any terms attached to the material and/or to the CBD.

Kew will supply the material listed on the reverse of this agreement ("Material") subject to the following terms and conditions:

1. The recipient may only use the Material, its progeny or derivatives for the common good in **scientific research, education, conservation and the development of botanic gardens**;
2. The recipient shall **not sell, distribute or use for profit or any other commercial application**[1] the Material, its progeny or derivatives;
3. The recipient shall **share fairly and equitably** the benefits arising from their use of the Material, its progeny or derivatives in accordance with the CBD. You will find a non exhaustive list of non-monetary and monetary benefits at Appendix II to the Bonn Guidelines: www.biodiv.org/programmes/socio-eco/benefit/bonn.asp;
4. The recipient shall **acknowledge** Kew, as supplier, in all written or electronic reports and publications resulting from their use of the Material, its progeny and derivatives and shall **lodge a copy** of all such publications and reports with Kew;
5. The recipient shall take **all appropriate and necessary measures** to import the Material in accordance with relevant laws and regulations and to contain the Material, its progeny or derivatives so as to prevent the release of invasive alien species;
6. The recipient may only **transfer** the Material, its progeny or derivatives to a bona fide third party such as a botanic garden, university or scientific institution for **non-commercial** use in the areas of scientific research, education, conservation and the development of botanic gardens. All transfers shall be subject to the terms and conditions of this agreement. The recipient shall **notify Kew** of all such transfers and, on request, shall provide Kew with copies of the relevant material transfer agreement;
7. The recipient shall maintain **retrievable records** linking the Material to these terms of acquisition and to any accompanying Data provided by Kew;
8. Unless otherwise indicated, **copyright** in all information or data ("Data") supplied with the Material is owned by Kew or Kew's licensors. You may use this Data on condition that it is used solely for scholarly, education or research purposes; that it is not used for commercial purposes; and that you always acknowledge the source of the Data with the words "With the permission of the Board of Trustees of the Royal Botanic Gardens, Kew";
9. Kew makes **no representation or warranty** of any kind, either express or implied, as to the identity, safety, merchantability or fitness for any particular purpose of the Material, its progeny or derivatives, or as to the accuracy or reliability of any Data supplied. The recipient will indemnify Kew from any and all liability arising from the Material, its progeny or derivatives and/or the Data and from their use or transfer, including any ecological damage. This agreement is governed by and shall be construed in accordance with English law;
10. The recipient will contact Kew to request **prior permission** from Kew or, where appropriate, from the provider of the Material to Kew, for any activities not covered under the terms of this agreement.

I agree to comply with the conditions above: 20061109 & 20061110

Signed: L Galloway	Date: dd/mm/yy 15/08/06
Name and Position: LOUISE GALLOWAY SUPERVISOR GLASS DEPT.	Organisation and Department: ROYAL BOTANIC GARDEN EDINBURGH
Address: R.B.G.E. 20A INVERLEITH ROW EDINBURGH, EH3 5LR	E-mail: l-galloway@rbge.org.uk<br>Tel. Number: 0131-248-2919

Please return a signed copy to: Nick Johnston GGT,
Royal Botanic Gardens, Kew, Richmond Surrey TW9 3AE, United Kingdom.

Kew Staff Signature:	Name/Position/Date: dd/mm/yy:

[1] For the purposes of this agreement, commercial application shall mean: applying for, obtaining or transferring intellectual property rights or other tangible or intangible rights by sale or licence or in any other manner; commencement of product development; conducting market research; seeking pre-market approval; and/or the sale of any resulting product.

Royal Botanic Garden Edinburgh

Conditions of use of material derived from the RBGE Living Collection

1. In response to the Convention on Biological Diversity, the Royal Botanic Garden Edinburgh supplies living plant material, preserved material, seed, DNA and pollen samples on condition that:

a) The material is used for the common good in areas of Research, Education, Conservation and the development of Botanic Gardens.
b) Access to material from RBGE's collections is for non-commercial use only. If the recipient seeks to commercialise the genetic material, its products or resources derived from it, then written permission must be sought from the RBGE. Such commercialism will be subject to the conditions of a separate agreement.
c) The genetic material, its products or resources deriving from it are not passed on to a third party for commercialisation without written permission from the country of origin and the Royal Botanic Garden Edinburgh in accordance with the Convention on Biological Diversity.

2. It is a condition of supply that any publication resulting from the use of plant material should acknowledge the Royal Botanic Garden Edinburgh as supplier or, under certain circumstances, be published in collaboration with staff from RBGE. A copy of any publication, report or data gained from the material MUST be lodged with the Royal Botanic Garden Edinburgh.

4. A voucher specimen will be taken for material used for scientific purposes. This specimen will be kept at the RBGE and will be released on a loan basis. The cultivated collector and number must be referred to when requesting the voucher specimen and citing the material, not the accession number or the wild collector and number.

5. An aliquot from each DNA sampling will be returned to the RBGE.

6. Nagoya Protocol Compliance: from 12th October 2014 plant material requested from RBGE may be subject to Prior Informed Consent from the country of origin. Any permissions and conditions of use will be advised before release and if there are any restrictions it will not be made available. RBGE reserves the right to refuse, without explanation if necessary, to supply genetic resources to a recipient.

7. The Royal Botanic Garden Edinburgh cannot guarantee the material as verified.

Please return this form to:
Peter Brownless.
Royal Botanic Garden Edinburgh
20A Inverleith Row,Edinburgh ,EH3 5LR
Scotland, UK.
Fax: +44 (0)131 248 2901
Email attachment as a JPEG or Pdf to p.brownless@rbge.org.uk

For official use	
Internal contact:	
Desiderata number:	
Psource number:	
CBD DS#	
Other	

I agree to comply with the conditions above.

NAME	Sarah O'Connor	Position of Representative:	Project leader/Professor
Organisation:	John Innes Centre		
Department:	Biological Chemistry		
Address:	Norwich research park		
Post/Zip code:	NR4 7UH		
Tel. Number:	01603 450334		
Email:	Sarah.oconnor@jic.ac.uk	Country:	UK
Signed:	Sarah O'Connor	Fax. Number:	

Please retain a copy of this form for your own records

Compliance with the terms of the Nagoya protocol

The *Nagoya Protocol on access to genetic resources and the fair and equitable sharing of benefits arising from their utilization to the Convention on Biological Diversity* is a supplementary to Convention on Biological Diversity (CBD) and was adopted by European legislation on 13th October 2014.

The Nagoya Protocol provides a transparent legal framework for the implementation of fair and equitable sharing of benefits arising out of the utilization of genetic resources. The Protocol applies to all genetic resources covered by the CBD, the traditional knowledge associated with those resources and the benefits arising from the utilization of those resources.

The Protocol sets out three types of obligations:

Access obligations include providing fair and non-arbitrary procedures for access, establishing clear procedures for prior informed consent and creating conditions to promote and encourage research.

Benefit-sharing obligations include measures to provide for the fair and equitable sharing of benefits arising from the utilisation of genetic resources. Utilization includes research and development on the genetic or biochemical composition of genetic resources, as well as subsequent applications and commercialisation.

Compliance obligations include ensuring genetic resources have been accessed in accordance with prior informed consent and monitoring the utilization of genetic resources.

Oxford University Herbaria, Botanic Garden and Harcourt Arboretum (OHBGHA) intend to honour the letter and spirit of the Nagoya Protocol:

1. All material accessed to collections after 13th October 2014 will require evidence it has been collected legally, with the consent of the originating country. Copies of all evidence will be associated with each accession record.

2. All material accessed after 13th October 2014 will be tagged as such in electronic databases and its use restricted until appropriate agreements have been signed.

3. Ethnobotanical data, associated with material accessed under the Nagoya Protocol, via web-based databases will be restricted to registered database users who have signed appropriate agreements.

4. All use of material in OHBGHA under the Nagoya Protocol will be recorded. Agreement to the terms of the Protocol will be obtained by signing the form 'Collections Access Agreement for University of Oxford Herbaria, Botanic Garden & Harcourt Arboretum'.

For additional information regarding the Nagoya Protocol: CBD (2011) *Nagoya Protocol on Access to Genetic Resources and the Fair and Equitable Sharing of Benefits Arising from their Utilization to the Convention on Biological Diversity*. Secretariat of the Convention on Biological Diversity; Darwin Initiative (2014) *Learning note: the Nagoya Protocol*. Department for Environment, Food & Rural Affairs; Greiber, T. *et al.* (2012) *An explanatory guide to the Nagoya Protocol on access and benefit-sharing*. IUCN Environmental Policy and Law Paper No. 83.
